# Differential temperature control in heat-integrated pressure-swing distillation for separating azeotropes to deal with operating pressure fluctuations: Basic and explanatory data

**DOI:** 10.1016/j.dib.2020.105937

**Published:** 2020-06-26

**Authors:** Yongchao He, Ye Li, Kaitian Zheng, Guoning Li, Zheng Guo, Chunjian Xu

**Affiliations:** School of Chemical Engineering and Technology, Chemical Engineering Research Center and State Key Laboratory of Chemical Engineering, Tianjin University, Tianjin 300350, China

**Keywords:** Pressure-swing distillation, Heat-integrated, Differential temperature control, Binary azeotropes

## Abstract

This data article contains basic and explanatory data of “Differential temperature control in heat-integrated pressure-swing distillation for separating azeotropes to deal with operating pressure fluctuations” [1], including thermodynamic models, vapor-liquid equilibrium diagrams, steady-state flowsheets, temperature profiles of columns, description and setpoint units of the abbreviation of inventory control loops, description of control loops of control flowsheet with DTC, control flowsheets of PCTC and DTC, temperature and composition tuning constants, faceplates and flowsheet equations, dynamic performances and integral absolute errors. It also contains other important information.

**Specifications Table**

**Table:** 

Subject	Chemical Engineering (General)
Specific subject area	Control of distillation processes for separating azeotropes
Type of data	Table Image Graph Figure
How data were acquired	Aspen Plus and Aspen Dynamics
Data format	Raw Processed
Parameters for data collection	To make an overall assessment, the selected cases covered different important elements of HI-PSD, including minimum/maximum-boiling azeotropes, PHI/FHI-PSD, LPC->HPC/HPC->LPC sequence and conventional/entrainer-assisted PSD.
Description of data collection	The data were collected by simulations with Aspen Plus and Aspen Dynamics.
Data source location	Primary data sources: (1) Z. Zhu, L. Wang, Y. Ma, W. Wang, Y, Wang, Separating an azeotropic mixture of toluene and ethanol via heat integration pressure swing distillation, Comput. Chem. Eng. 76 (2015) 137–149. (2) W. L. Luyben, Comparison of extractive distillation and pressure-swing distillation for acetone-methanol separation, Ind. Eng. Chem. Res. 47 (2008) 2696–2707. (3) E. Hosgor, T. Kucuk, I. N. Oksal, D. B. Kaymak, Design and control of distillation processes for methanol-chloroform separation, Comput. Chem. Eng. 67 (2014) 166–177. (4) Q. Zhang, M. Liu, C. Li, A. Zeng, Heat-integrated pressure-swing distillation process for separating the minimum-boiling azeotrope ethyl-acetate and ethanol, Sep. Purif. Technol. 189 (2017) 310–334. (5) Y. Li, W. Li, L. Zhong, C. Xu, Entrainer-assisted pressure-swing distillation for separating the minimum-boiling azeotrope toluene/pyridine: design and control, Ind. Eng. Chem. Res. 56 (2017) 11,894–11,902. (6) W. L. Luyben, Methanol/trimethoxysilane azeotrope separation using pressure-swing distillation, Ind. Eng. Chem. Res. 53 (2014) 5590–5597. (7) W. L. Luyben, Control of a heat-integrated pressure-swing distillation process for the separation of a maximum-boiling azeotrope, Ind. Eng. Chem. Res. 53 (2014) 18,042–18,053.
Data accessibility	With the article
Related research article	Y. He, Y. Li, K. Zheng, G. Li, Z. Guo, C. Xu, Differential temperature control in heat-integrated pressure-swing distillation for separating azeotropes to deal with operating pressure fluctuations, Appl. Therm. Eng. 178 (2020) In Press.

**Value of the Data**

• The data provide the insights on simulations of steady-state design and dynamic control of the investigated processes, including full information of thermodynamic models, vapor-liquid equilibrium diagrams, steady-state flowsheets, temperature profiles of columns, description and setpoint units of the abbreviation of inventory control loops, description of control loops of control flowsheet with DTC, control flowsheets of PCTC and DTC, temperature and composition tuning constants, faceplates and flowsheet equations, dynamic performances and integral absolute errors.
• The data will be useful for the related researchers to understand how the simulations are established.
• The data provide a baseline for exploring other potential aspects of DTC in future.

## 1 Data description

Table 1 shows thermodynamic models of all the mixtures.

**Table 1:** Thermodynamic models of all the mixtures.

mixture	thermodynamic model^a^
toluene/ethanol	NRTL
acetone/methanol	UNIQUAC
methanol/chloroform	NRTL
ethanol/ethyl acetate	UNIQUAC
toluene/pyridine	NRTL
methanol/trimethoxysilane	UNIFAC

a The default parameters for the chosen thermodynamic models are used.

Table 2 shows the description and setpoint units of the abbreviation of inventory control loops of the control faceplate.

**Table 2:** The description and setpoint units of the abbreviation of inventory control loops of the control faceplate.

abbreviation	description^a^	unit of set point
FC	fresh feed flow rate controller	kmol/h or kg/h
DC (if it has)	1st column distillate flow rate controller	kmol/h
D2C (if it has)	2nd column distillate flow rate controller	kmol/h
B1C (if it has)	1st column bottom flow rate controller	kmol/h
B2C (if it has)	2nd column bottom flow rate controller	kmol/h
PC1	1st column pressure controller	bar
PC2	2nd column pressure controller	bar
LC1	1st column reflux drum level controller	m
LC2	1st column sump level controller	m
LC3	2nd column reflux drum level controller	m
LC4	2nd column sump level controller	m

a The 1st column represents the left column in the process flowsheet and the 2nd column represents the right column in the process flowsheet.

Table 3 shows controller tuning constants of the proposed control structure with DTC of PHI-PSD for separating the toluene/ethanol mixture.

**Table 3:** Controller tuning constants of the proposed control structure with DTC of PHI-PSD for separating the toluene/ethanol mixture.

Controlled Variable	Manipulated Variable	Gain *K*_C_ (%/%)	Integral Time *τ*_I_ (min)	Derivative Time *τ*_D_ (min)
T21	*Q*_Aux_	37.45	7.92	–
CC	T21	29.35	75.24	
DT	*Q*_R2_	0.44	7.92	–

Table 4 shows controller tuning constants of the proposed control structure with PCTC of PHI-PSD for separating the toluene/ethanol mixture.

**Table 4:** Controller tuning constants of the proposed control structure with PCTC of PHI-PSD for separating the toluene/ethanol mixture.

Controlled Variable	Manipulated Variable	Gain *K*_C_ (%/%)	Integral Time *τ*_I_ (min)	Derivative Time *τ*_D_ (min)
T21	*Q*_Aux_	37.45	7.92	–
CC	T21	29.35	75.24	
T20	*Q*_R2_	1.39	10.56	–

Table 5 shows controller tuning constants of the proposed control structure with DTC of PHI-PSD for separating the acetone/methanol mixture.

**Table 5:** Controller tuning constants of the proposed control structure with DTC of PHI-PSD for separating the acetone/methanol mixture.

Controlled Variable	Manipulated Variable	Gain *K*_C_ (%/%)	Integral Time *τ*_I_ (min)	Derivative Time *τ*_D_ (min)
T47	*Q*_Aux_	11.94	7.92	–
DT	*Q*_R2_	0.80	9.24	–

Table 6 shows controller tuning constants of the proposed control structure with PCTC of PHI-PSD for separating the acetone/methanol mixture.

**Table 6:** Controller tuning constants of the proposed control structure with PCTC of PHI-PSD for separating the acetone/methanol mixture.

Controlled Variable	Manipulated Variable	Gain *K*_C_ (%/%)	Integral Time *τ*_I_ (min)	Derivative Time *τ*_D_ (min)
T47	*Q*_Aux_	11.94	7.92	–
T55	*Q*_R2_	30.95	9.24	–

Table 7 shows controller tuning constants of the proposed control structure with DTC of FHI-PSD for separating the toluene/ethanol mixture.

**Table 7:** Controller tuning constants of the proposed control structure with DTC of FHI-PSD for separating the toluene/ethanol mixture.

Controlled Variable	Manipulated Variable	Gain *K*_C_ (%/%)	Integral Time *τ*_I_ (min)	Derivative Time *τ*_D_ (min)
T11	*RR*_1_	53.45	25.08	–
DT	*Q*_R2_	0.45	7.92	–

Table 8 shows controller tuning constants of the proposed control structure with PCTC of FHI-PSD for separating the toluene/ethanol mixture.

**Table 8:** Controller tuning constants of the proposed control structure with PCTC of FHI-PSD for separating the toluene/ethanol mixture.

Controlled Variable	Manipulated Variable	Gain *K*_C_ (%/%)	Integral Time *τ*_I_ (min)	Derivative Time *τ*_D_ (min)
T11	*RR*_1_	53.45	25.08	–
T19	*Q*_R2_	1.39	10.56	–

Table 9 shows controller tuning constants of the proposed control structure with DTC of FHI-PSD for separating the methanol/chloroform mixture.

**Table 9:** Controller tuning constants of the proposed control structure with DTC of FHI-PSD for separating the methanol/chloroform mixture.

Controlled Variable	Manipulated Variable	Gain *K*_C_ (%/%)	Integral Time *τ*_I_ (min)	Derivative Time *τ*_D_ (min)
T19	*RR*_1_	7.22	25.08	–
DT	*Q*_R2_	0.35	7.92	–

Table 10 shows controller tuning constants of the proposed control structure with PCTC of FHI-PSD for separating the methanol/chloroform mixture.

**Table 10:** Controller tuning constants of the proposed control structure with PCTC of FHI-PSD for separating the methanol/chloroform mixture.

Controlled Variable	Manipulated Variable	Gain *K*_C_ (%/%)	Integral Time *τ*_I_ (min)	Derivative Time *τ*_D_ (min)
T19	*RR*_1_	7.22	25.08	–
T23	*Q*_R2_	2.47	9.24	–

Table 11 shows controller tuning constants of the proposed control structure with DTC of FHI-PSD for separating the ethyl acetate/ethanol mixture.

**Table 11:** Controller tuning constants of the proposed control structure with DTC of FHI-PSD for separating the ethyl acetate/ethanol mixture.

Controlled Variable	Manipulated Variable	Gain *K*_C_ (%/%)	Integral Time *τ*_I_ (min)	Derivative Time *τ*_D_ (min)
T25	*RR*_2_	3.96	27.72	–
DT	*Q*_R1_	0.39	10.56	–

Table 12 shows controller tuning constants of the proposed control structure with PCTC of FHI-PSD for separating the ethyl acetate/ethanol mixture.

**Table 12:** Controller tuning constants of the proposed control structure with PCTC of FHI-PSD for separating the ethyl acetate/ethanol mixture.

Controlled Variable	Manipulated Variable	Gain *K*_C_ (%/%)	Integral Time *τ*_I_ (min)	Derivative Time *τ*_D_ (min)
T25	*RR*_2_	3.96	27.72	–
T50	*Q*_R1_	9.01	9.24	–

Table 13 shows controller tuning constants of the proposed control structure with DTC of EA-PSD for separating the toluene/pyridine mixture.

**Table 13:** Controller tuning constants of the proposed control structure with DTC of EA-PSD for separating the toluene/pyridine mixture.

Controlled Variable	Manipulated Variable	Gain *K*_C_ (%/%)	Integral Time *τ*_I_ (min)	Derivative Time *τ*_D_ (min)
T16	*Q*_Aux_	28.30	10.56	–
DT	*Q*_R2_	0.53	7.92	–
CC	DF	50.73	432.96	–

Table 14 shows controller tuning constants of the proposed control structure with PCTC of EA-PSD for separating the toluene/pyridine mixture.

**Table 14:** Controller tuning constants of the proposed control structure with PCTC of EA-PSD for separating the toluene/pyridine mixture.

Controlled Variable	Manipulated Variable	Gain *K*_C_ (%/%)	Integral Time *τ*_I_ (min)	Derivative Time *τ*_D_ (min)
T16	*Q*_Aux_	28.30	10.56	–
T18	*Q*_R2_	7.69	9.24	–
CC	DF	50.73	432.96	–

Table 15 shows controller tuning constants of the proposed control structure with DTC of the HPC->LPC of PHI-PSD for separating the methanol/trimethoxysilane mixture.

**Table 15:** Controller tuning constants of the proposed control structure with DTC of the HPC->LPC of PHI-PSD for separating the methanol/trimethoxysilane mixture.

Controlled Variable	Manipulated Variable	Gain *K*_C_ (%/%)	Integral Time *τ*_I_ (min)	Derivative Time *τ*_D_ (min)
DT	*RR*_1_	0.23	11.88	–
T5	1/*RR*_2_	14.13	50.16	–

Table 16 shows controller tuning constants of the proposed control structure with PCTC of the HPC->LPC of PHI-PSD for separating the methanol/trimethoxysilane mixture.

**Table 16:** Controller tuning constants of the proposed control structure with PCTC of the HPC->LPC of PHI-PSD for separating the methanol/trimethoxysilane mixture.

Controlled Variable	Manipulated Variable	Gain *K*_C_ (%/%)	Integral Time *τ*_I_ (min)	Derivative Time *τ*_D_ (min)
T3	*RR*_1_	2.23	13.20	–
T5	1/*RR*_2_	13.96	50.16	–

Table 17 shows controller tuning constants of the proposed control structure with DTC of the LPC->HPC of PHI-PSD for separating the methanol/trimethoxysilane mixture.

**Table 17:** Controller tuning constants of the proposed control structure with DTC of the LPC->HPC of PHI-PSD for separating the methanol/trimethoxysilane mixture.

Controlled Variable	Manipulated Variable	Gain *K*_C_ (%/%)	Integral Time *τ*_I_ (min)	Derivative Time *τ*_D_ (min)
DT	*RR*_1_	1.99	11.88	–
CC	DT	14.88	34.32	
DT2	1/*RR*_2_	0.33	50.16	–

Table 18 shows controller tuning constants of the proposed control structure with PCTC of the LPC->HPC of PHI-PSD for separating the methanol/trimethoxysilane mixture.

**Table 18:** Controller tuning constants of the proposed control structure with PCTC of the LPC->HPC of PHI-PSD for separating the methanol/trimethoxysilane mixture.

Controlled Variable	Manipulated Variable	Gain *K*_C_ (%/%)	Integral Time *τ*_I_ (min)	Derivative Time *τ*_D_ (min)
DT	*RR*_1_	1.99	11.88	–
CC	DT	14.69	34.32	
T3	1/*RR*_2_	3.16	48.84	–

Table 19 shows Comparison of IAE of important variables between PCTC and DTC under large feed disturbances.

**Table 19:** Comparison of IAE of important variables between PCTC and DTC under large feed disturbances.

Process	Control type	Item	Unit	*F* + 20%	F-20%	*C* + 20%	C-20%	Sum
toluene and ethanol Min Azeotrope PHI LPC->HPC	PCTC	ethanol	10^^−4^ kg/kg	–	4.26	0.74*	0.27	5.28
		toluene	10^^−4^ kg/kg	–	53.12	8.14*	7.06	68.32
		QR (HPC)	kW	–	6236.7	2310.7*	71.3	8618.8
		T 20	°C	–	349.3	122.6*	29.7	501.6
	DTC	ethanol	10^^−4^ kg/kg	–	4.27	0.74*	0.27	5.28
		toluene	10^^−4^ kg/kg	–	52.05	5.23*	10.83	68.11
		QR (HPC)	kW	–	6249.3	2319.5*	65.5	8634.2
		D T	°C	–	7.29	0.99*	0.42	8.70
acetone and methanol Min Azeotrope PHI LPC->HPC	PCTC	methanol	10^^−3^ mol/mol	–	41.22	1.12	–	42.34
		acetone	10^^−3^ mol/mol	–	24.55	30.06	–	54.61
		QR (HPC)	kW	–	28,928.4	18,540.1	–	47,468.4
		T 55	°C	–	210.6	146.6	–	357.2
	DTC	methanol	10^^−3^ mol/mol	–	41.58	1.13	–	42.71
		acetone	10^^−3^ mol/mol	–	75.27	32.34	–	107.61
		QR (HPC)	kW	–	30,160.5	18,559.0	–	48,719.4
		D T	°C	–	0.22	0.06	–	0.28
toluene and ethanol Min Azeotrope FHI LPC->HPC	PCTC	ethanol	10^^−3^ kg/kg	–	22.58	19.00*	133.00	174.58
		toluene	10^^−3^ kg/kg	–	3.99	0.78*	1.39	6.16
		QR (HPC)	kW	–	5300.8	2245.4*	2466.7	10,012.9
		T 19	°C	–	273.4	106.9*	108.8	489.1
	DTC	ethanol	10^^−3^ kg/kg	–	22.59	19.00*	132.78	174.37
		toluene	10^^−3^ kg/kg	–	3.68	0.42*	0.34	4.44
		QR (HPC)	kW	–	5316.8	2248.9*	2470.9	10,036.7
		D T	°C	–	5.75	0.70*	1.09	7.54
methanol and chloroform Min Azeotrope FHI LPC->HPC	PCTC	methanol	10^^−3^ mol/mol	–	–	29.77	26.68	56.46
		chloroform	10^^−3^ mol/mol	–	–	11.12	4.02	15.14
		QR (HPC)	kW	–	–	792.2	3019.2	3811.4
		T 23	°C	–	–	18.9	71.1	90.0
	DTC	methanol	10^^−3^ mol/mol	–	–	29.83	26.63	56.46
		chloroform	10^^−3^ mol/mol	–	–	15.28	3.52	18.80
		QR (HPC)	kW	–	–	790.8	3035.1	3825.8
		D T	°C	–	–	1.49	0.30	1.79
ethanol and ethyl acetate Min Azeotrope FHI HPC->LPC	PCTC	ethyl acetate	10^^−3^ mol/mol	15.14	15.37	15.42	15.26	61.20
		ethanol	10^^−3^ mol/mol	53.69	35.43	24.47	30.00	143.59
		QR (HPC)	kW	12,428.6	11,640.4	400.4	308.8	24,778.2
		T 50	°C	223.0	195.4	38.8	47.6	504.7
	DTC	ethyl acetate	10^^−3^ mol/mol	11.82	17.32	18.64	16.47	64.24
		ethanol	10^^−3^ mol/mol	53.12	36.96	24.63	30.11	144.82
		QR (HPC)	kW	12,265.4	12,820.5	309.9	358.7	25,754.5
		D T	°C	0.24	0.31	0.28	0.23	1.06
toluene and pyridine Min Azeotrope EA	PCTC	pyridine	10^^−3^ mol/mol	4.51*	6.96	1.16	1.16	13.79
		toluene	10^^−3^ mol/mol	6.42*	4.20	5.72	4.53	20.87
		QR (HPC)	kW	12,557.5*	16,806.6	800.2	808.8	30,973.0
		T 18	°C	200.8*	249.1	12.0	12.2	474.2
	DTC	pyridine	10^^−3^ mol/mol	4.95*	6.96	1.16	1.16	14.23
		toluene	10^^−3^ mol/mol	8.22*	4.28	5.68	4.49	22.67
		QR (HPC)	kW	12,518.2*	16,804.5	799.3	807.7	30,929.7
		D T	°C	0.78*	1.15	0.04	0.05	2.02
methanol and trimethoxysilane Max Azeotrope PHI HPC->LPC	PCTC	methanol	10^^−2^ mol/mol	14.40	4.98	17.76	12.97	50.11
		trimethoxysilane	10^^−2^ mol/mol	11.92	9.50	8.02	11.01	40.45
		RR1	–	2.21	8.91	33.72	62.26	107.10
		T 3	°C	548.2	273.4	315.6	607.0	1744.2
	DTC	methanol	10^^−2^ mol/mol	7.48	5.47	18.55	12.38	43.87
		trimethoxysilane	10^^−2^ mol/mol	12.30	9.52	8.05	11.21	41.07
		RR1	–	4.74	8.86	33.78	63.64	111.02
		D T	°C	1.21	1.77	6.25	10.11	19.35
methanol and trimethoxysilane Max Azeotrope PHI LPC->HPC	PCTC	methanol	10^^−2^ mol/mol	16.20	8.41	27.54	13.50	65.65
		trimethoxysilane	10^^−2^ mol/mol	0.86	0.69	0.57	0.38	2.50
		1/RR2	–	0.17	0.24	0.39	0.03	0.84
		T 3	°C	593.7	312.8	771.1	376.2	2053.8
	DTC	methanol	10^^−2^ mol/mol	7.98	9.09	9.53	11.45	38.05
		trimethoxysilane	10^^−2^ mol/mol	0.85	0.67	0.59	0.37	2.49
		1/RR2	–	0.26	0.24	0.52	0.04	1.05
		D T2	°C	3.82	7.12	5.32	6.42	22.68

*These values are the results of the corresponding 15% feed disturbances.

Table 20 shows ratio of reboiler duty to feed flow rate.

**Table 20:** Ratio of reboiler duty to feed flow rate.

Process	Ratio	Unit	
		Reboiler duty	Feed flow rate
PHI-PSD for separating the toluene/ethanol mixture	0.001404	MMkcal/hr	kg/hr
PHI-PSD for separating the acetone/methanol mixture	0.047812	MMkcal/hr	kmol/hr
FHI-PSD for separating the toluene/ethanol mixture	0.001565	MMkcal/hr	kg/hr
FHI-PSD for separating the methanol/chloroform mixture	0.068078	MMkcal/hr	kmol/hr
FHI-PSD for separating the ethanol/ethyl acetate mixture	0.106945	MMkcal/hr	kmol/hr
EA-PSD for separating the toluene/pyridine mixture	–	–	–
PHI-PSD for separating the methanol/trimethoxysilane mixture	0.100637	MMkcal/hr	kmol/hr
PHI-PSD for separating the methanol/trimethoxysilane mixture	0.061675	MMkcal/hr	kmol/hr

**Fig. 1:**
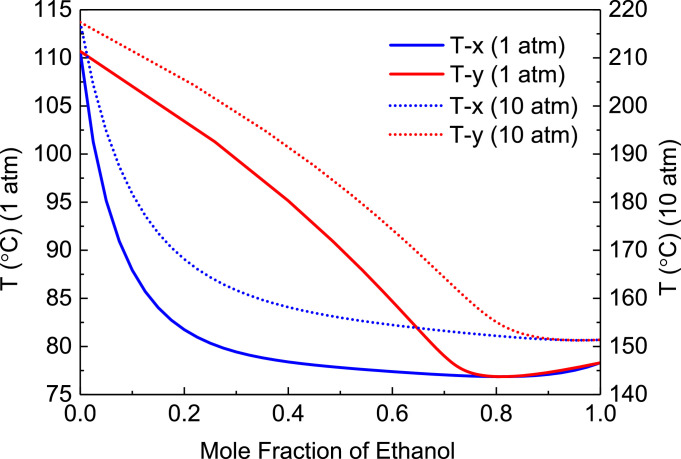
T-*xy* diagram of toluene/ethanol.

Fig. 1 shows T-xy diagram of toluene/ethanol.

**Fig. 2:**
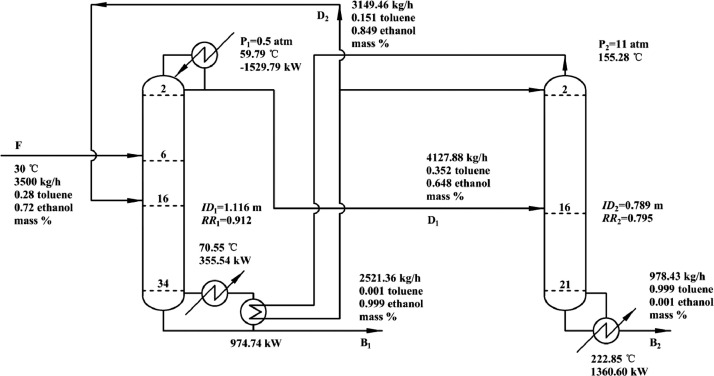
Flowsheet of PHI-PSD for separating the toluene/ethanol mixture.

Fig. 2 shows flowsheet of PHI-PSD for separating the toluene/ethanol mixture.

**Fig. 3:**
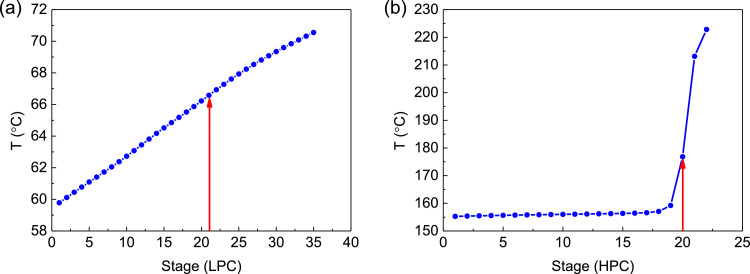
Temperature profiles of PHI-PSD for separating the toluene/ethanol mixture: (a) the LPC and (b) the HPC.

Fig. 3 shows temperature profiles of PHI-PSD for separating the toluene/ethanol mixture: (a) the LPC and (b) the HPC.

**Fig. 4:**
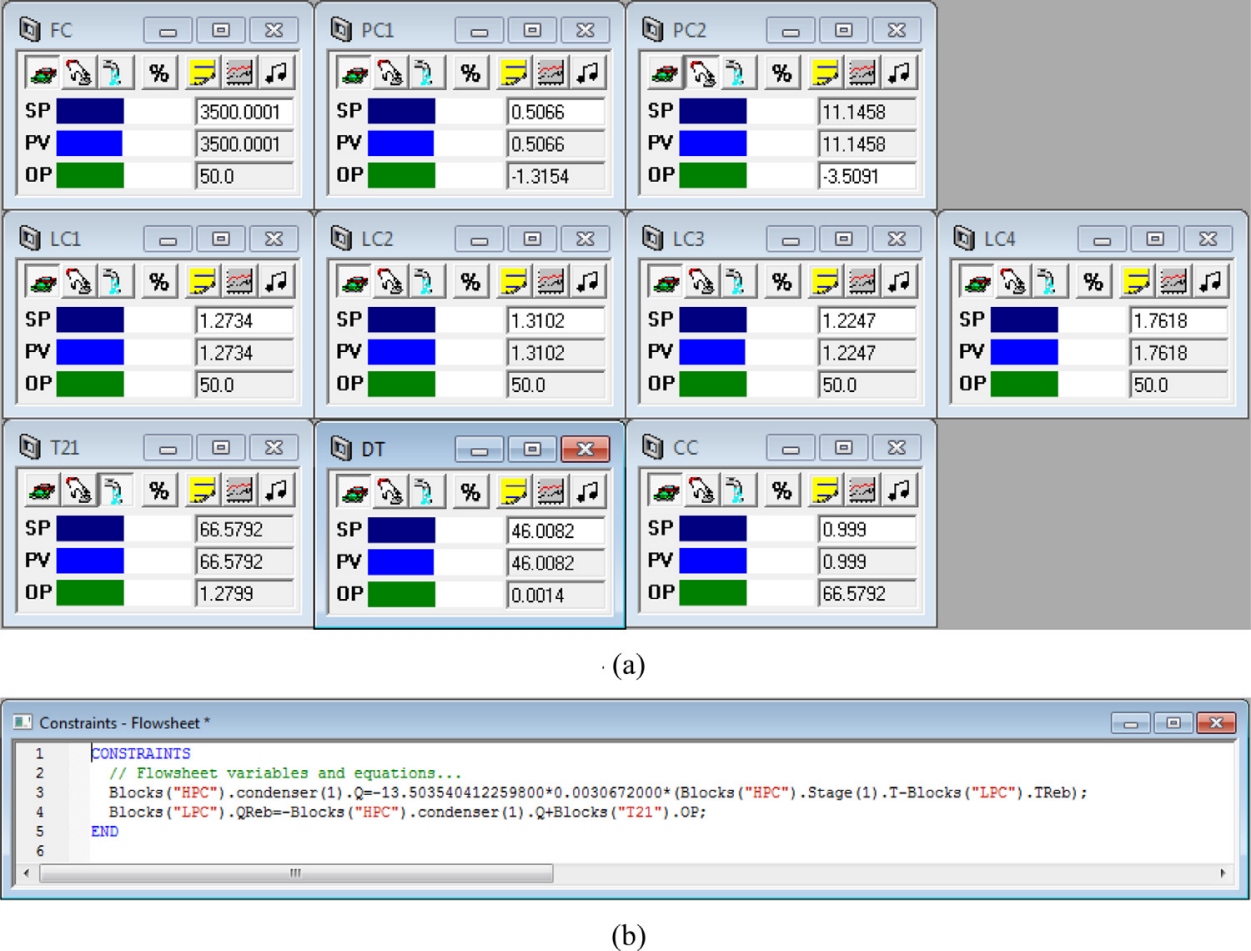
The proposed control structure with DTC of PHI-PSD for separating the toluene/ethanol mixture: (a) control faceplate and (b) flowsheet equations.

Fig. 4 shows the proposed control structure with DTC of PHI-PSD for separating the toluene/ethanol mixture: (a) control faceplate and (b) flowsheet equations.

**Fig. 5:**
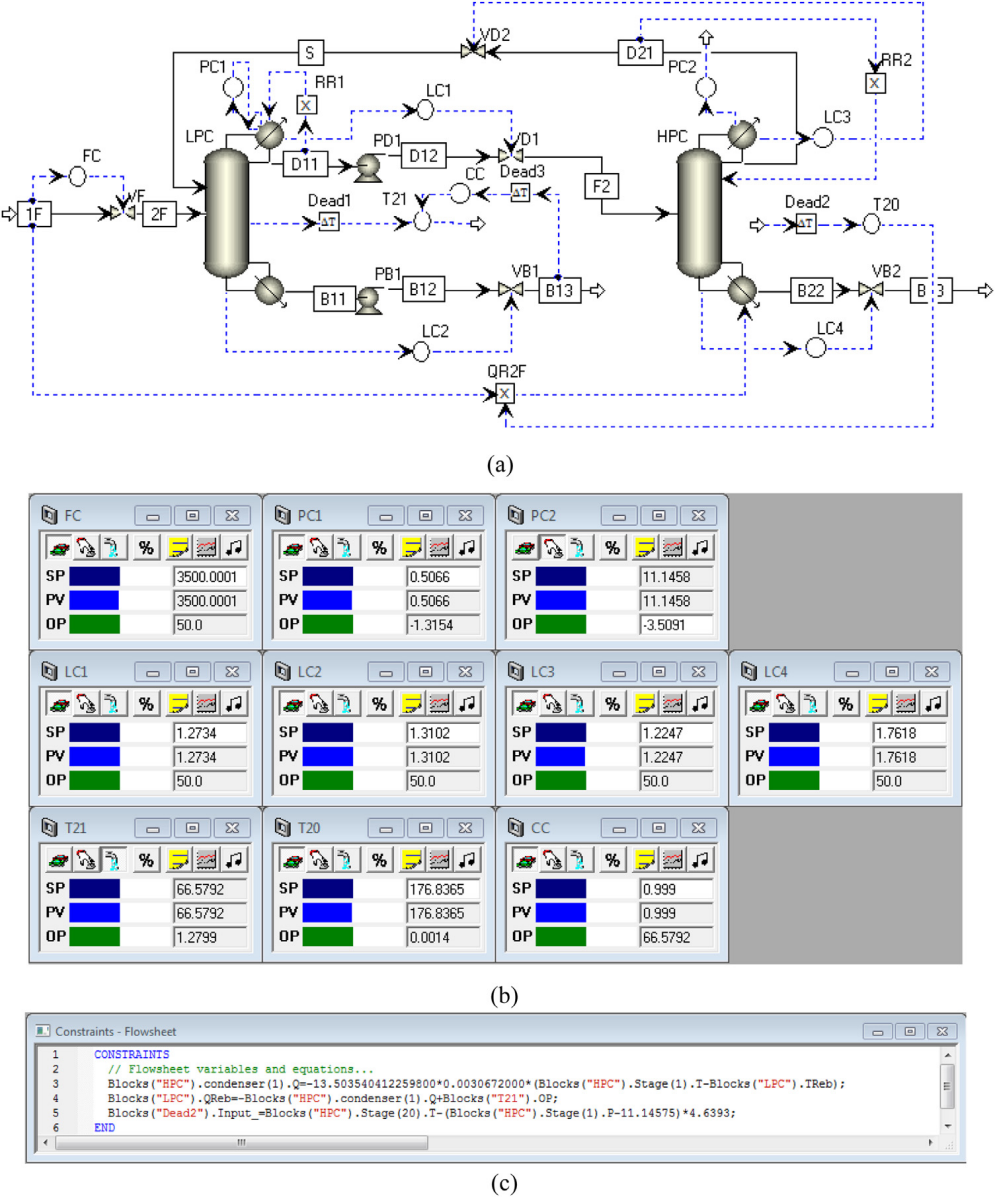
The proposed control structure with PCTC of PHI-PSD for separating the toluene/ethanol mixture: (a) control flowsheet, (b) control faceplate and (c) flowsheet equations.

Fig. 5 shows the proposed control structure with PCTC of PHI-PSD for separating the toluene/ethanol mixture: (a) control flowsheet, (b) control faceplate and (c) flowsheet equations.

**Fig. 6:**
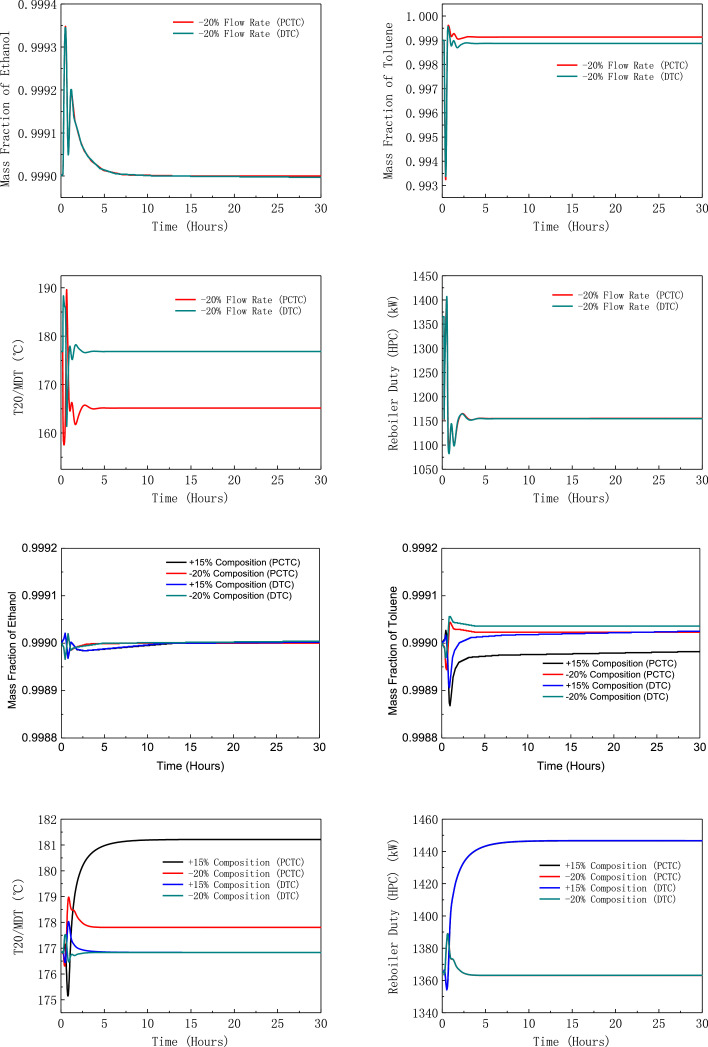
Dynamic performances of PHI-PSD for separating the toluene/ethanol mixture under large feed disturbances.

Fig. 6 shows dynamic performances of PHI-PSD for separating the toluene/ethanol mixture under large feed disturbances.

**Fig. 7:**
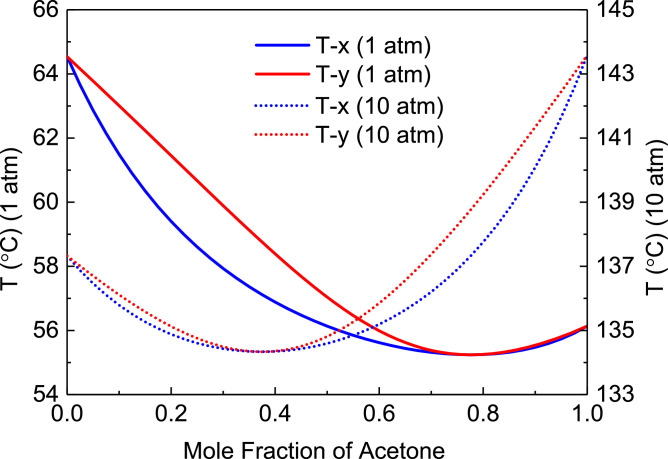
T-*xy* diagram of acetone/methanol.

Fig. 7 shows T-xy diagram of acetone/methanol.

**Fig. 8:**
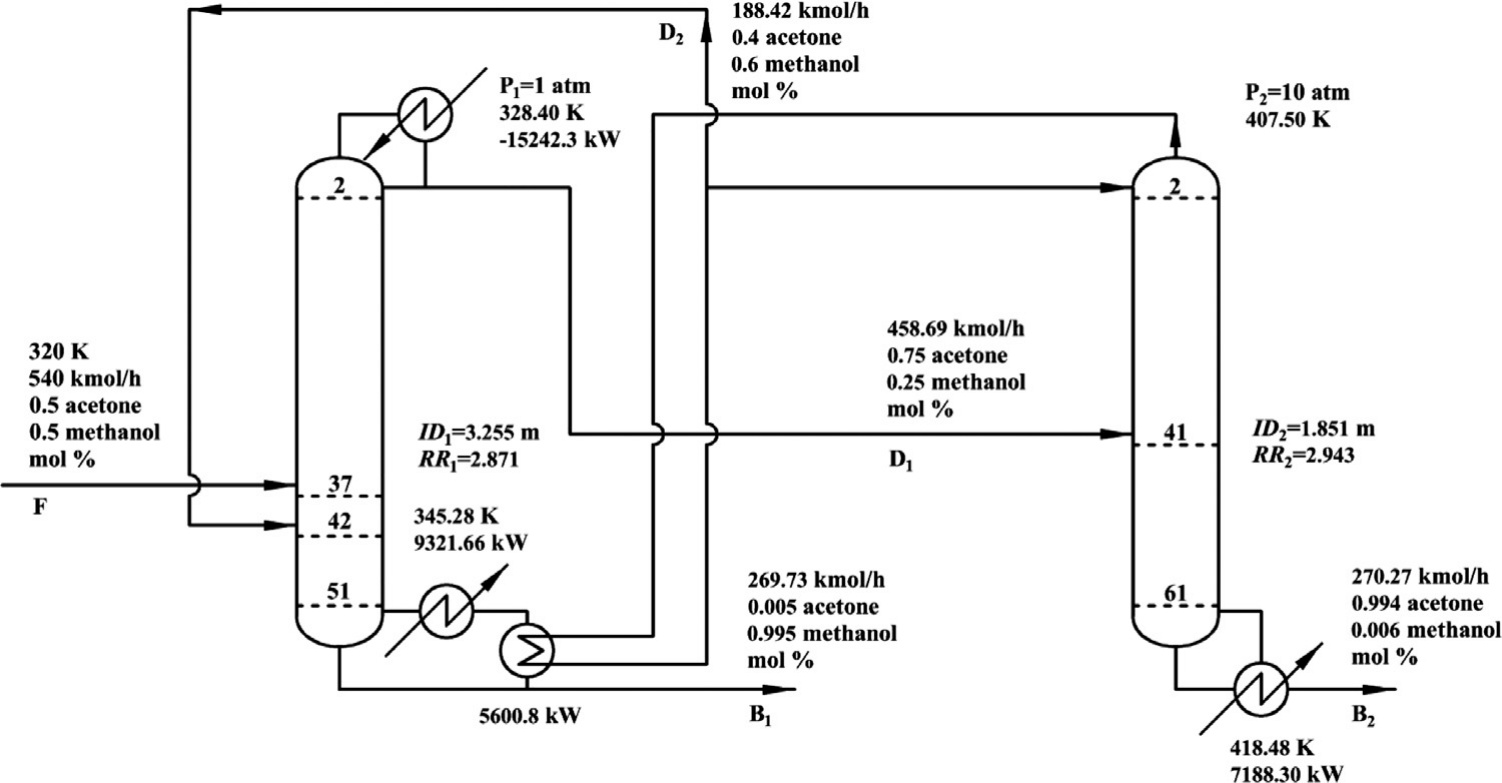
Flowsheet of PHI-PSD for separating the acetone/methanol mixture.

Fig. 8 shows flowsheet of PHI-PSD for separating the acetone/methanol mixture.

**Fig. 9:**
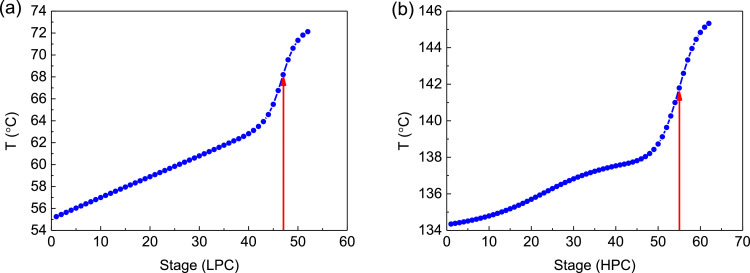
Temperature profiles PHI-PSD for separating the acetone/methanol mixture: (a) the LPC and (b) the HPC.

Fig. 9 shows temperature profiles PHI-PSD for separating the acetone/methanol mixture: (a) the LPC and (b) the HPC.

**Fig. 10:**
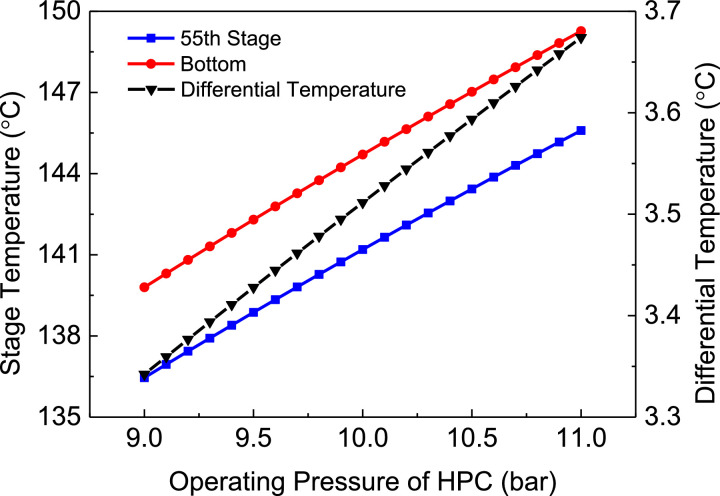
Differential temperature of PHI-PSD with DTC for separating the acetone/methanol mixture.

Fig. 10 shows differential temperature of PHI-PSD with DTC for separating the acetone/methanol mixture.

**Fig. 11:**
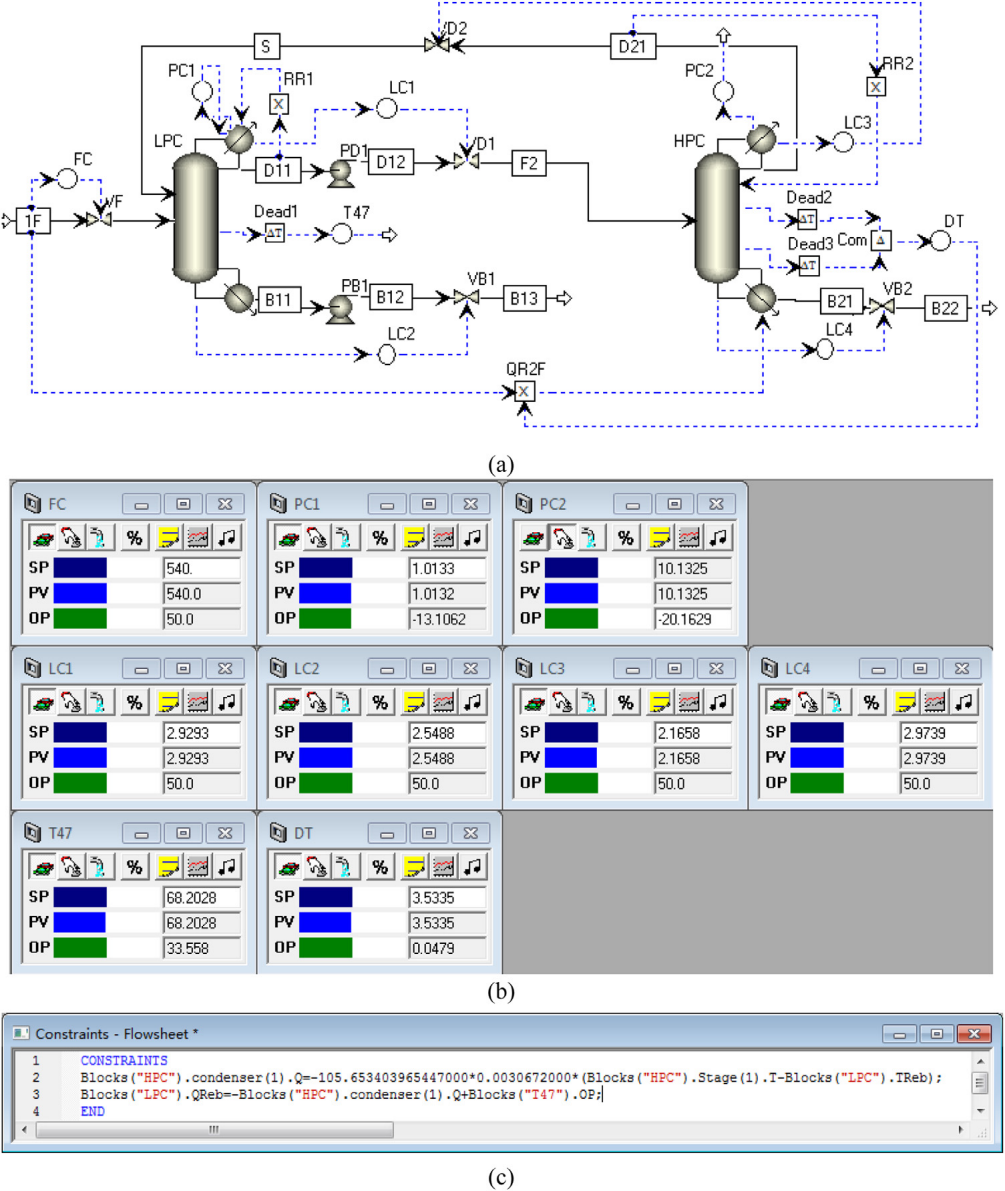
The proposed control structure with DTC of PHI-PSD for separating the acetone/methanol mixture: (a) control flowsheet, (b) control faceplate and (c) flowsheet equations.

Fig. 11 shows the proposed control structure with DTC of PHI-PSD for separating the acetone/methanol mixture: (a) control flowsheet, (b) control faceplate and (c) flowsheet equations.

**Fig. 12:**
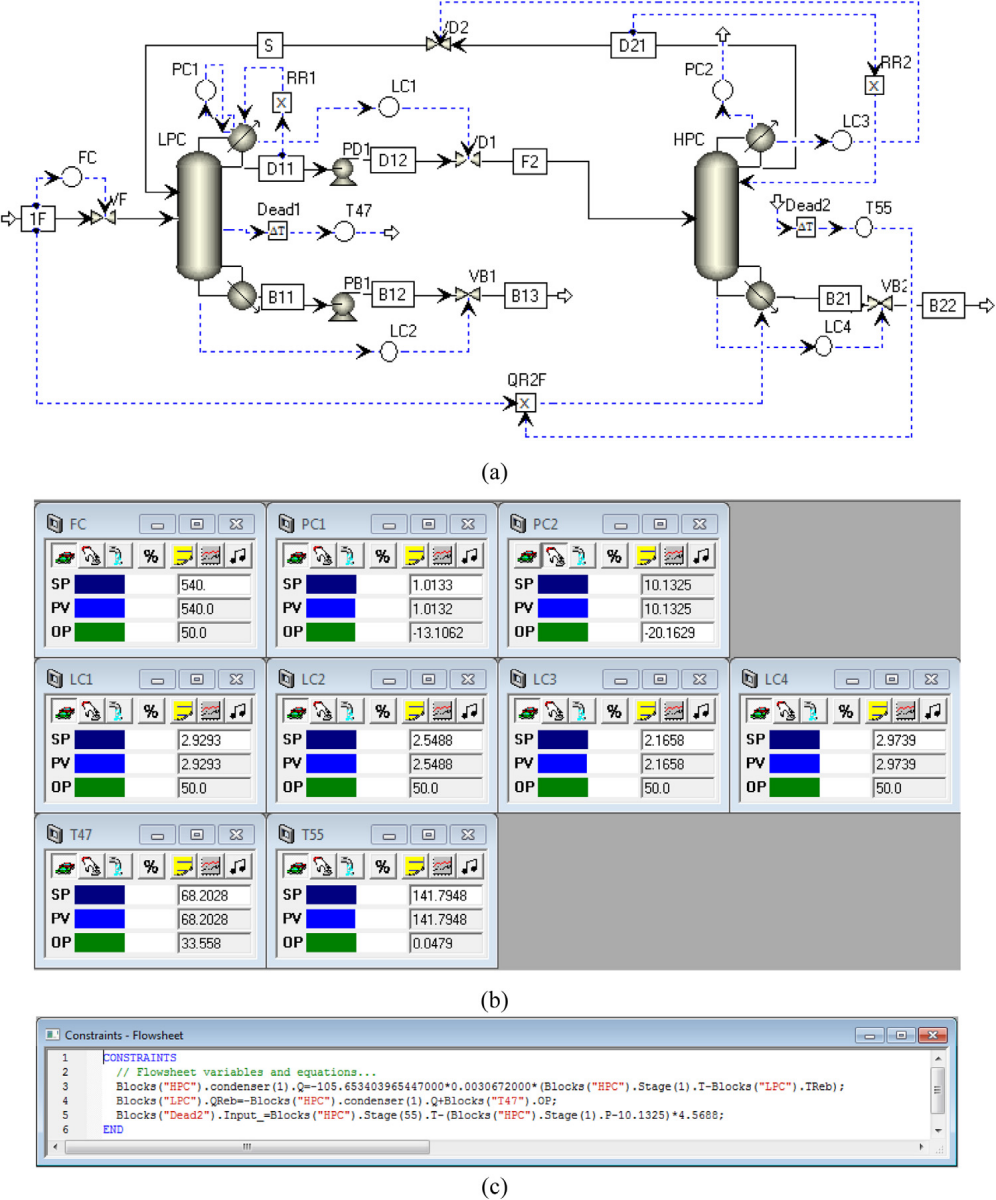
The proposed control structure with PCTC of PHI-PSD for separating the acetone/methanol mixture: (a) control flowsheet, (b) control faceplate and (c) flowsheet equations.

Fig. 12 shows the proposed control structure with PCTC of PHI-PSD for separating the acetone/methanol mixture: (a) control flowsheet, (b) control faceplate and (c) flowsheet equations.

**Fig. 13:**
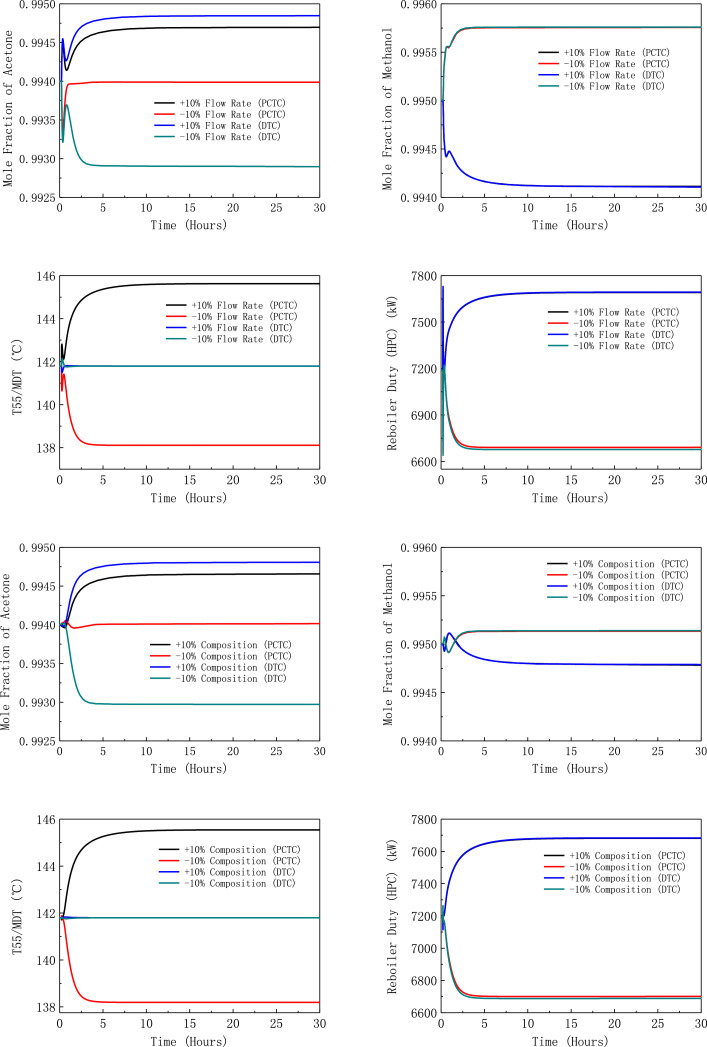
Dynamic performances of PHI-PSD for separating the acetone/methanol mixture under ±10% feed disturbances.

Fig. 13 shows dynamic performances of PHI-PSD for separating the acetone/methanol mixture under ±10% feed disturbances.

**Fig. 14:**
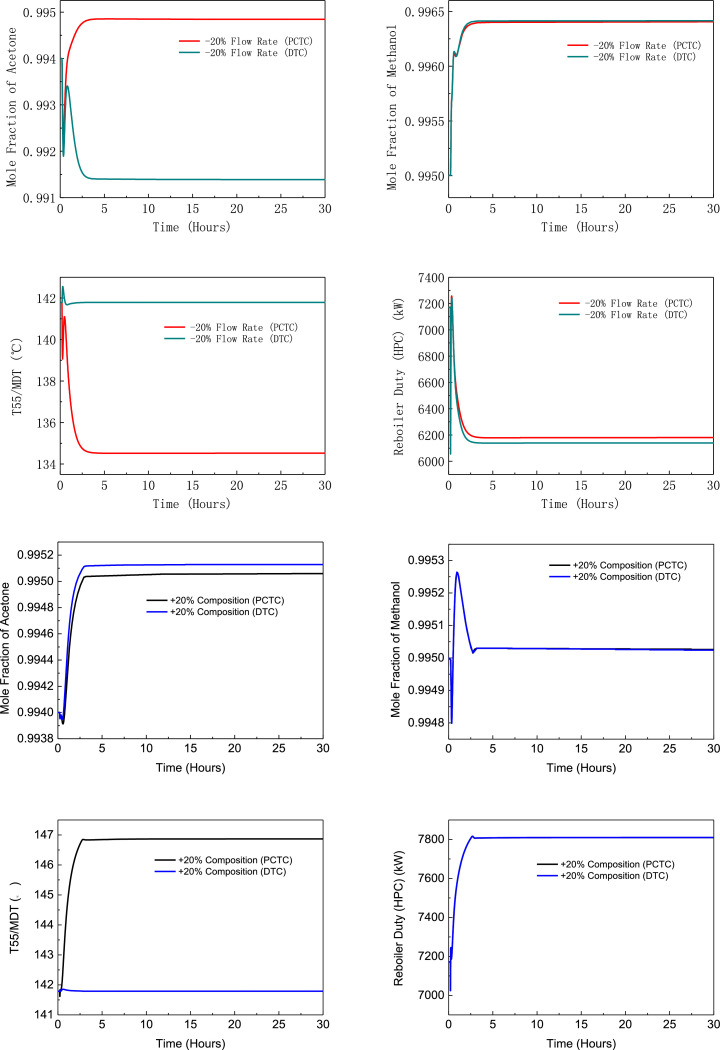
Dynamic performances of PHI-PSD for separating the acetone/methanol mixture under large feed disturbances.

Fig. 14 shows dynamic performances of PHI-PSD for separating the acetone/methanol mixture under large feed disturbances.

**Fig. 15:**
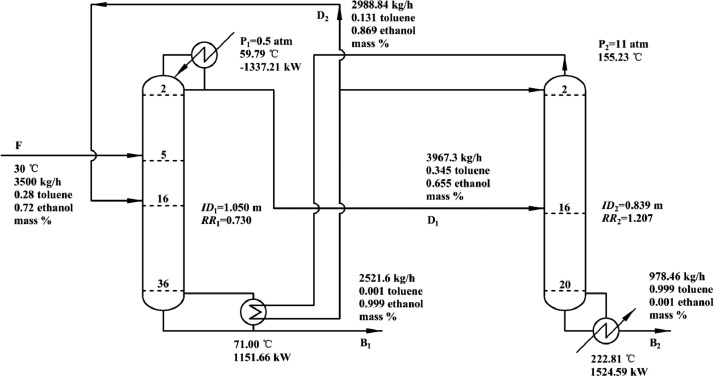
Flowsheet of FHI-PSD for separating the toluene/ethanol mixture.

Fig. 15 shows flowsheet of FHI-PSD for separating the toluene/ethanol mixture.

**Fig. 16:**
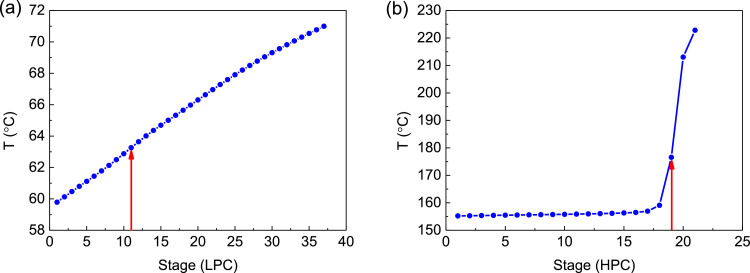
Temperature profiles of FHI-PSD for separating the toluene/ethanol mixture: (a) the LPC and (b) the HPC.

Fig. 16 shows temperature profiles of FHI-PSD for separating the toluene/ethanol mixture: (a) the LPC and (b) the HPC.

**Fig. 17:**
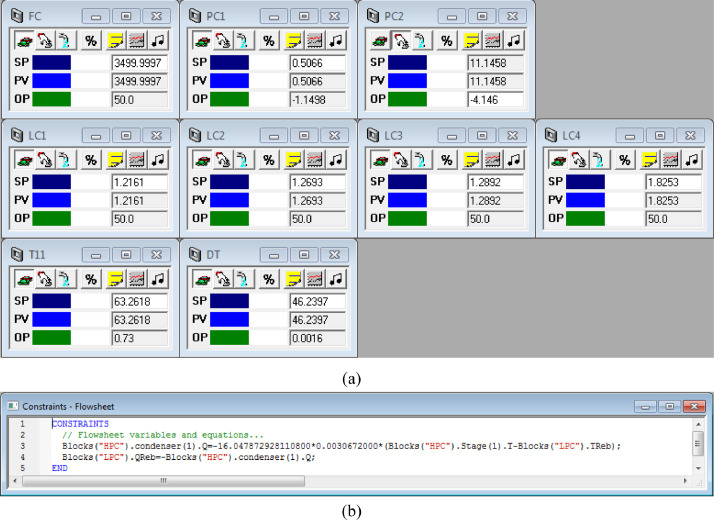
The proposed control structure with DTC of FHI-PSD for separating the toluene/ethanol mixture: (a) control faceplate and (b) flowsheet equations.

Fig. 17 shows the proposed control structure with DTC of FHI-PSD for separating the toluene/ethanol mixture: (a) control faceplate and (b) flowsheet equations.

**Fig. 18:**
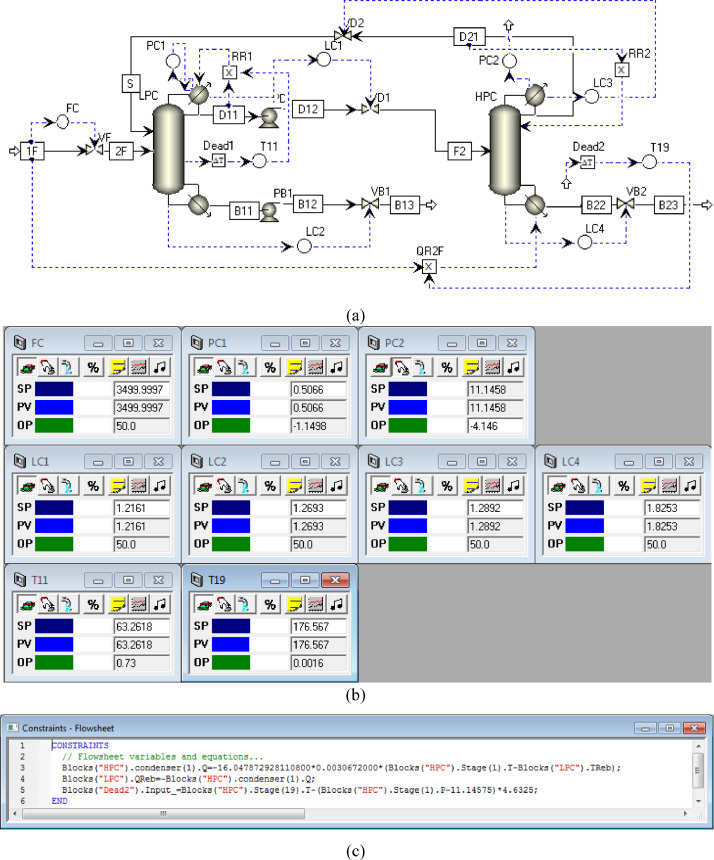
The proposed control structure with PCTC of FHI-PSD for separating the toluene/ethanol mixture: (a) control flowsheet, (b) control faceplate and (c) flowsheet equations.

Fig. 18 shows the proposed control structure with PCTC of FHI-PSD for separating the toluene/ethanol mixture: (a) control flowsheet, (b) control faceplate and (c) flowsheet equations.

**Fig. 19:**
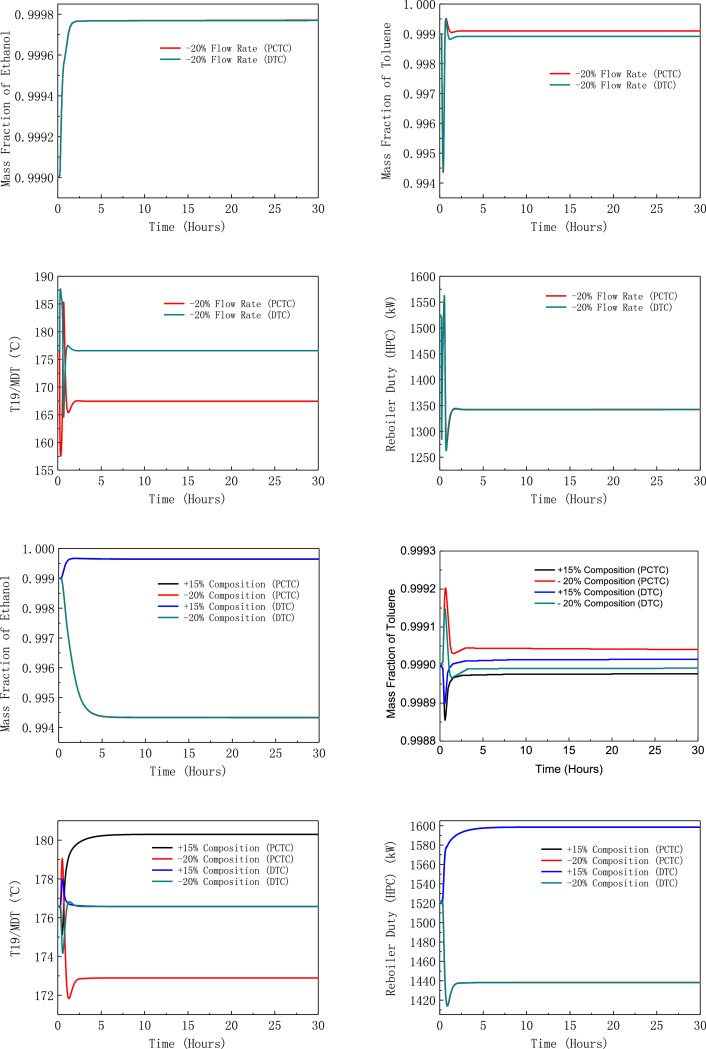
Dynamic performances of FHI-PSD for separating the toluene/ethanol mixture under large feed disturbances.

Fig. 19 shows dynamic performances of FHI-PSD for separating the toluene/ethanol mixture under large feed disturbances.

**Fig. 20:**
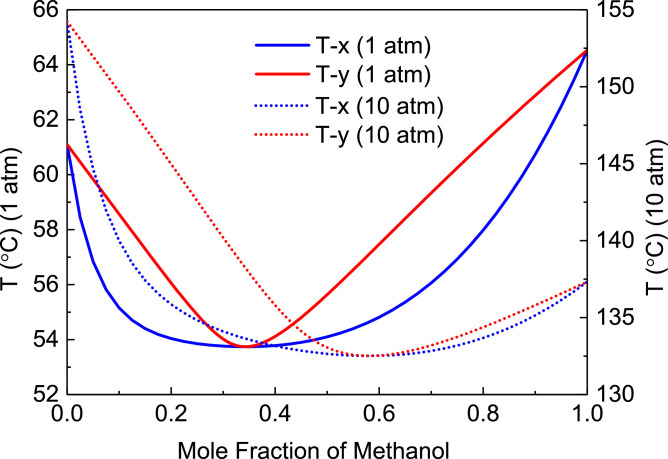
T-*xy* diagram of methanol/chloroform.

Fig. 20 shows T-xy diagram of methanol/chloroform.

**Fig. 21:**
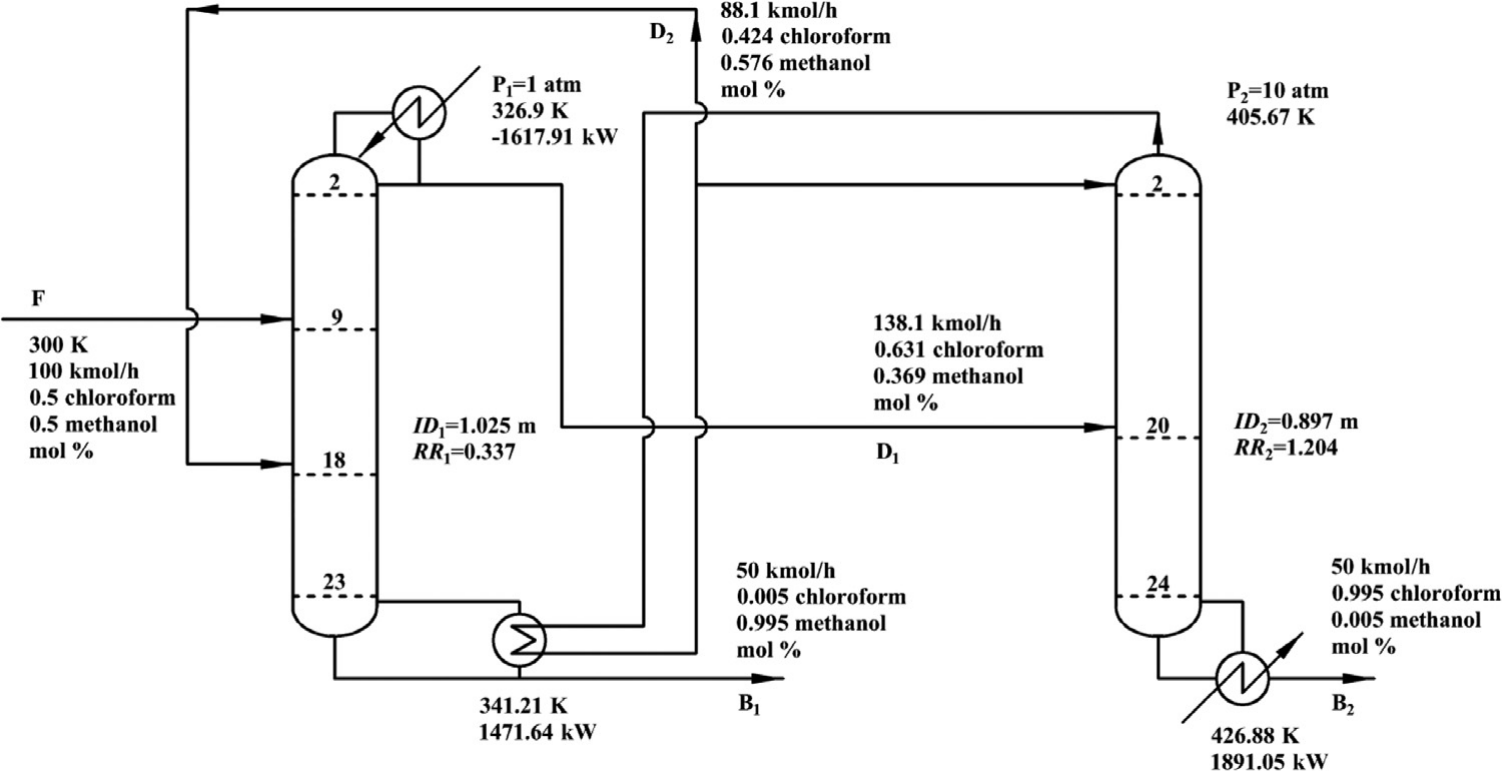
Flowsheet of FHI-PSD for separating the methanol/chloroform mixture.

Fig. 21 shows flowsheet of FHI-PSD for separating the methanol/chloroform mixture.

**Fig. 22:**
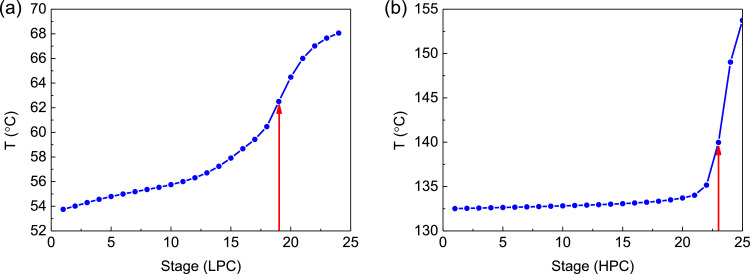
Temperature profiles of FHI-PSD for separating the methanol/chloroform mixture: (a) the LPC and (b) the HPC.

Fig. 22 shows temperature profiles of FHI-PSD for separating the methanol/chloroform mixture: (a) the LPC and (b) the HPC.

**Fig. 23:**
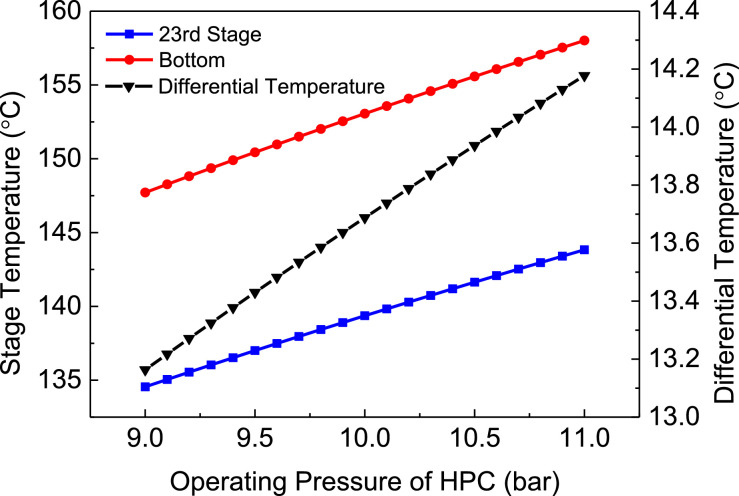
Differential temperature of FHI-PSD with DTC for separating the methanol/chloroform mixture.

Fig. 23 shows differential temperature of FHI-PSD with DTC for separating the methanol/chloroform mixture.

**Fig. 24:**
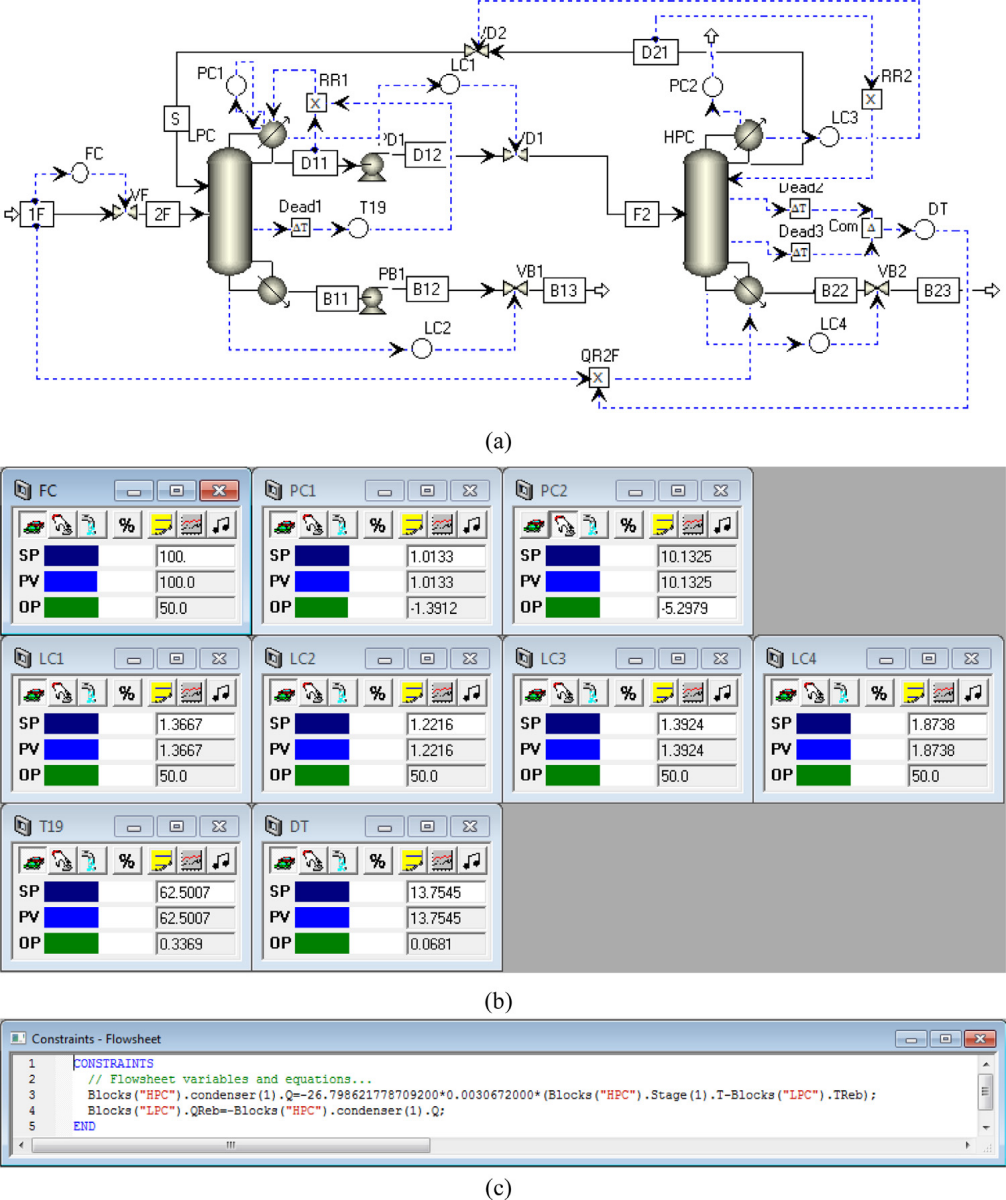
The proposed control structure with DTC of FHI-PSD for separating the methanol/chloroform mixture: (a) control faceplate and (b) flowsheet equations.

Fig. 24 shows the proposed control structure with DTC of FHI-PSD for separating the methanol/chloroform mixture: (a) control faceplate and (b) flowsheet equations.

**Fig. 25:**
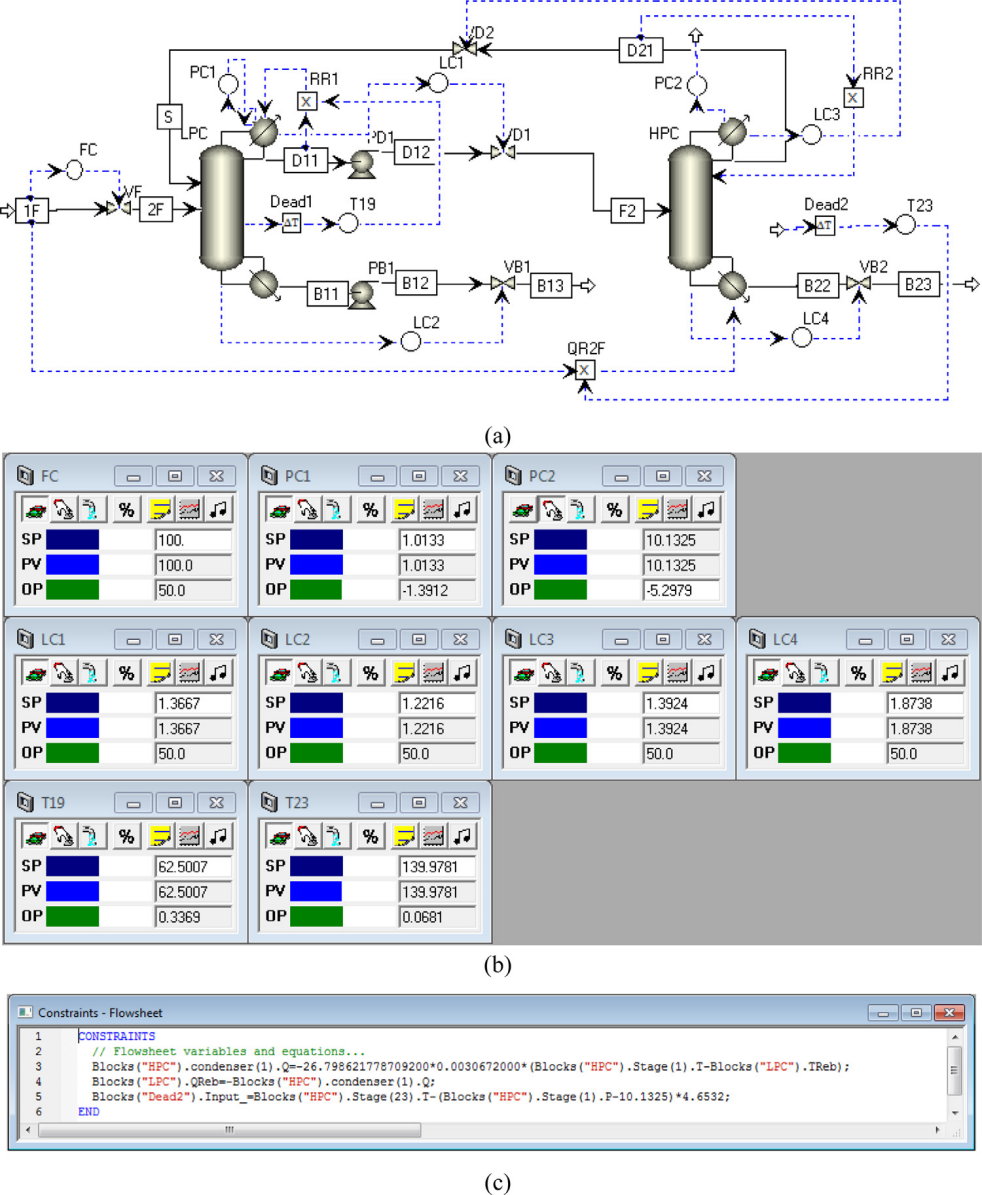
The proposed control structure with PCTC of FHI-PSD for separating the methanol/chloroform mixture: (a) control flowsheet, (b) control faceplate and (c) flowsheet equations.

Fig. 25 shows the proposed control structure with PCTC of FHI-PSD for separating the methanol/chloroform mixture: (a) control flowsheet, (b) control faceplate and (c) flowsheet equations.

**Fig. 26:**
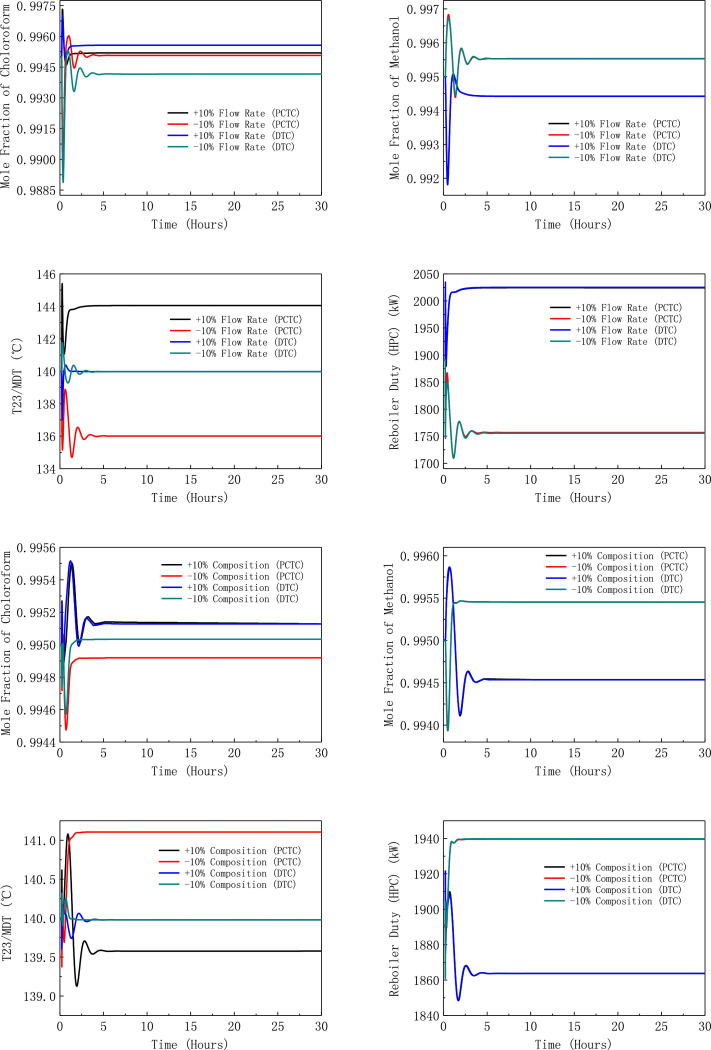
Dynamic performances of FHI-PSD for separating methanol/chloroform mixture under ±10% feed disturbances.

Fig. 26 shows dynamic performances of FHI-PSD for separating methanol/chloroform mixture under ±10% feed disturbances.

**Fig. 27:**
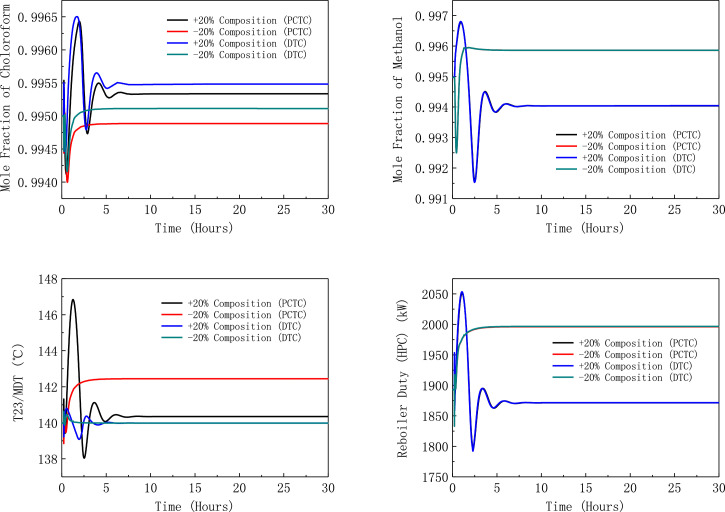
Dynamic performances of FHI-PSD for separating methanol/chloroform mixture under large feed disturbances.

Fig. 27 shows dynamic performances of FHI-PSD for separating methanol/chloroform mixture under large feed disturbances.

**Fig. 28:**
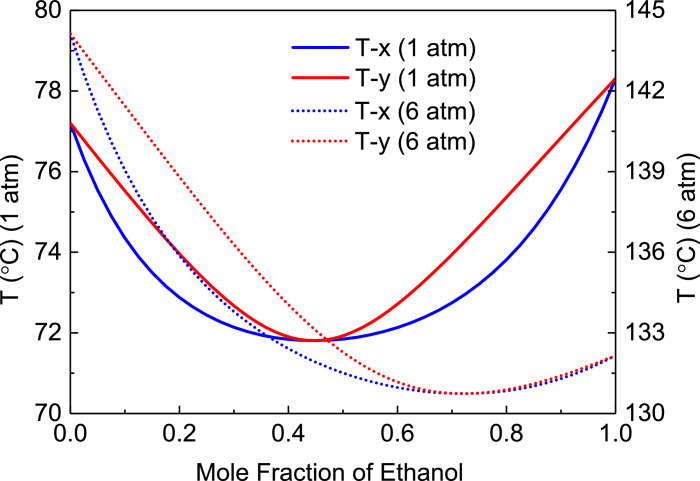
T-*xy* diagram of ethanol/ethyl acetate at 101.3 kPa.

Fig. 28 shows T-xy diagram of ethanol/ethyl acetate at 101.3 kPa.

**Fig. 29:**
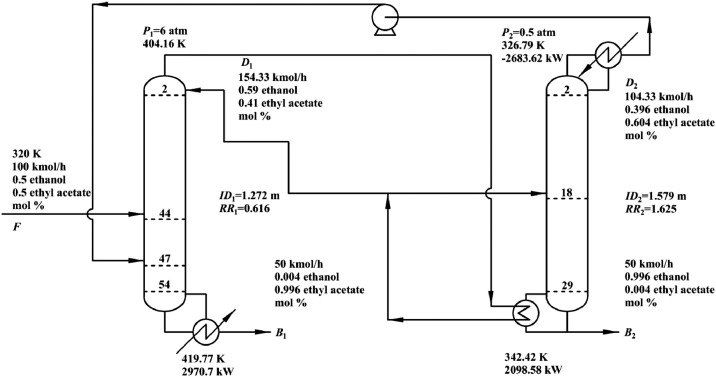
Flowsheet of FHI-PSD for separating the ethyl acetate/ethanol mixture.

Fig. 29 shows flowsheet of FHI-PSD for separating the ethyl acetate/ethanol mixture.

**Fig. 30:**
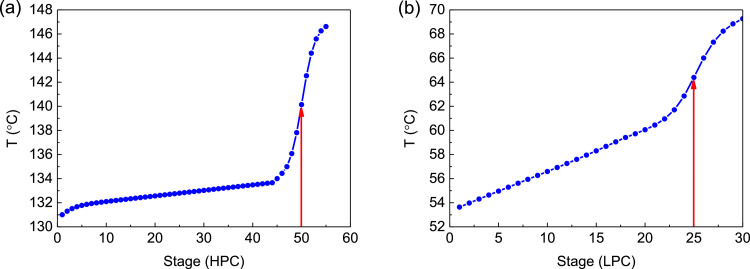
Temperature profiles of FHI-PSD for separating the ethyl acetate/ethanol mixture: (a) the HPC and (b) the LPC.

Fig. 30 shows temperature profiles of FHI-PSD for separating the ethyl acetate/ethanol mixture: (a) the HPC and (b) the LPC.

**Fig. 31:**
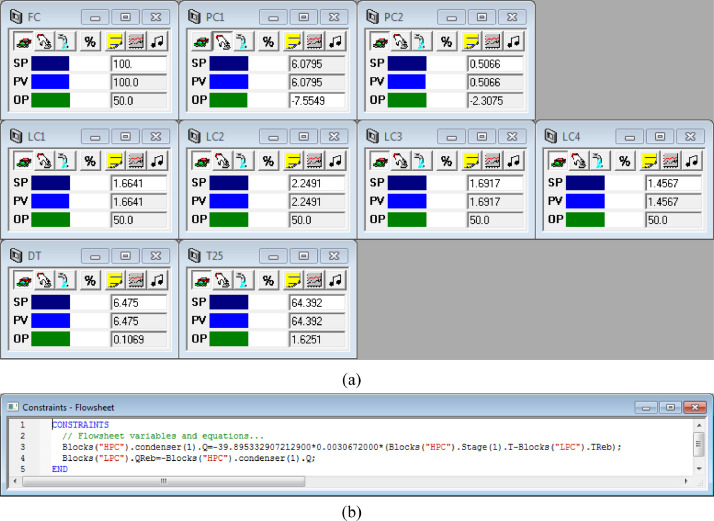
The proposed control structure with DTC of FHI-PSD for separating the ethyl acetate/ethanol mixture: (a) control faceplate and (b) flowsheet equations.

Fig. 31 shows the proposed control structure with DTC of FHI-PSD for separating the ethyl acetate/ethanol mixture: (a) control faceplate and (b) flowsheet equations.

**Fig. 32:**
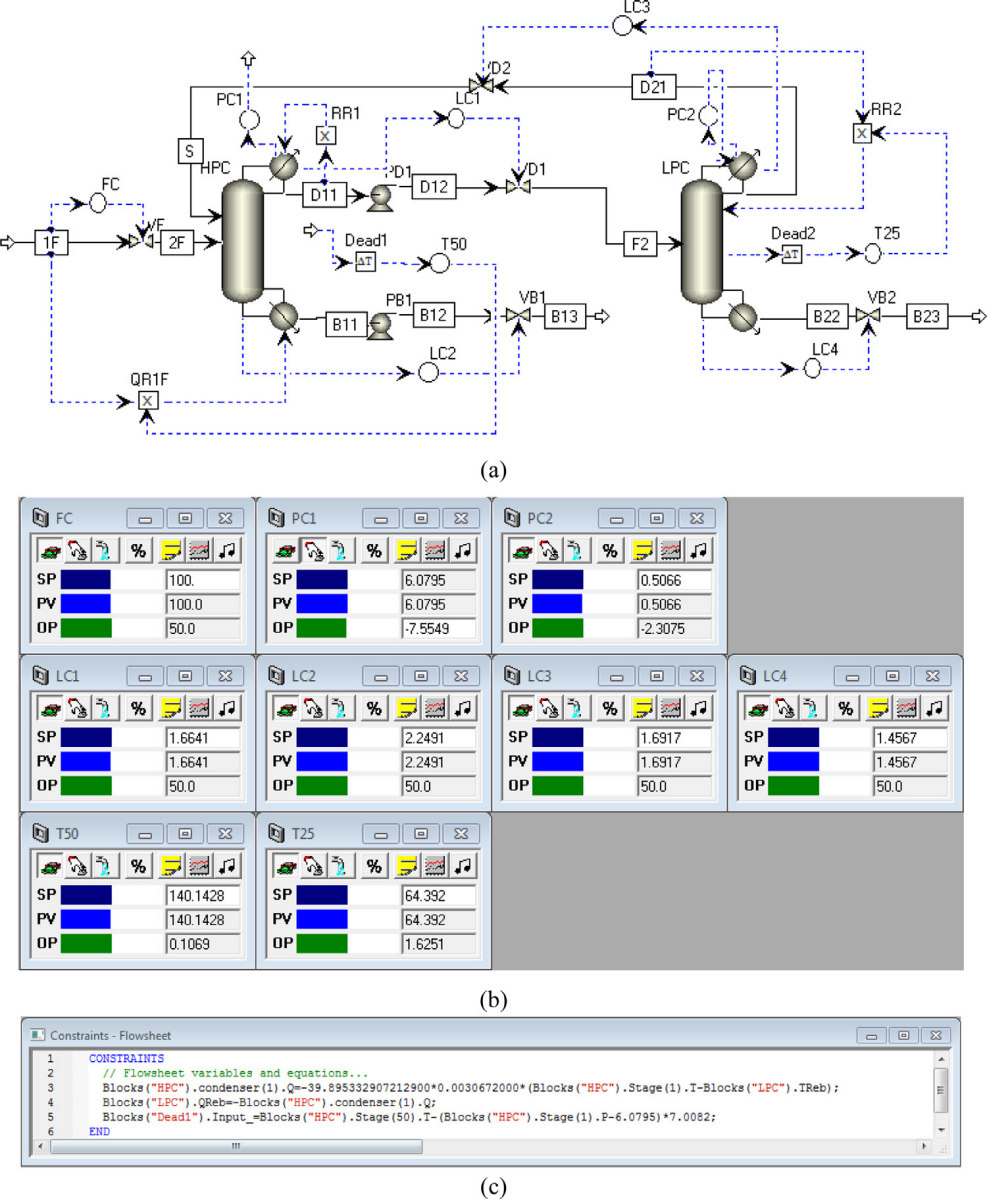
The proposed control structure with PCTC of FHI-PSD for separating the ethyl acetate/ethanol mixture: (a) control flowsheet, (b) control faceplate and (c) flowsheet equations.

Fig. 32 shows the proposed control structure with PCTC of FHI-PSD for separating the ethyl acetate/ethanol mixture: (a) control flowsheet, (b) control faceplate and (c) flowsheet equations.

**Fig. 33:**
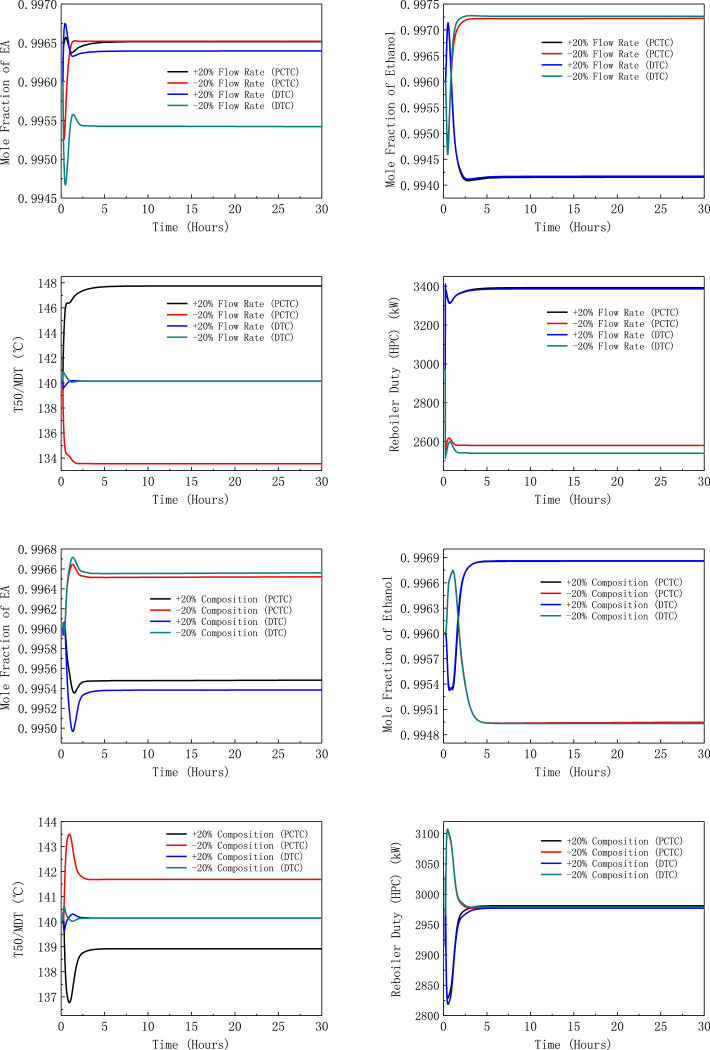
Dynamic performances of FHI-PSD for separating the ethanol/ethyl acetate mixture under large feed disturbances.

Fig. 33 shows dynamic performances of FHI-PSD for separating the ethanol/ethyl acetate mixture under large feed disturbances.

**Fig. 34:**
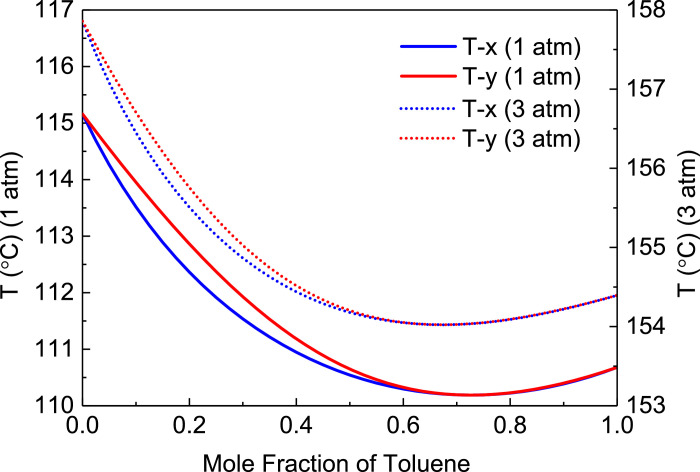
T-*xy* diagram of toluene/pyridine at 101.3 kPa.

Fig. 34 shows T-xy diagram of toluene/pyridine at 101.3 kPa.

**Fig. 35:**
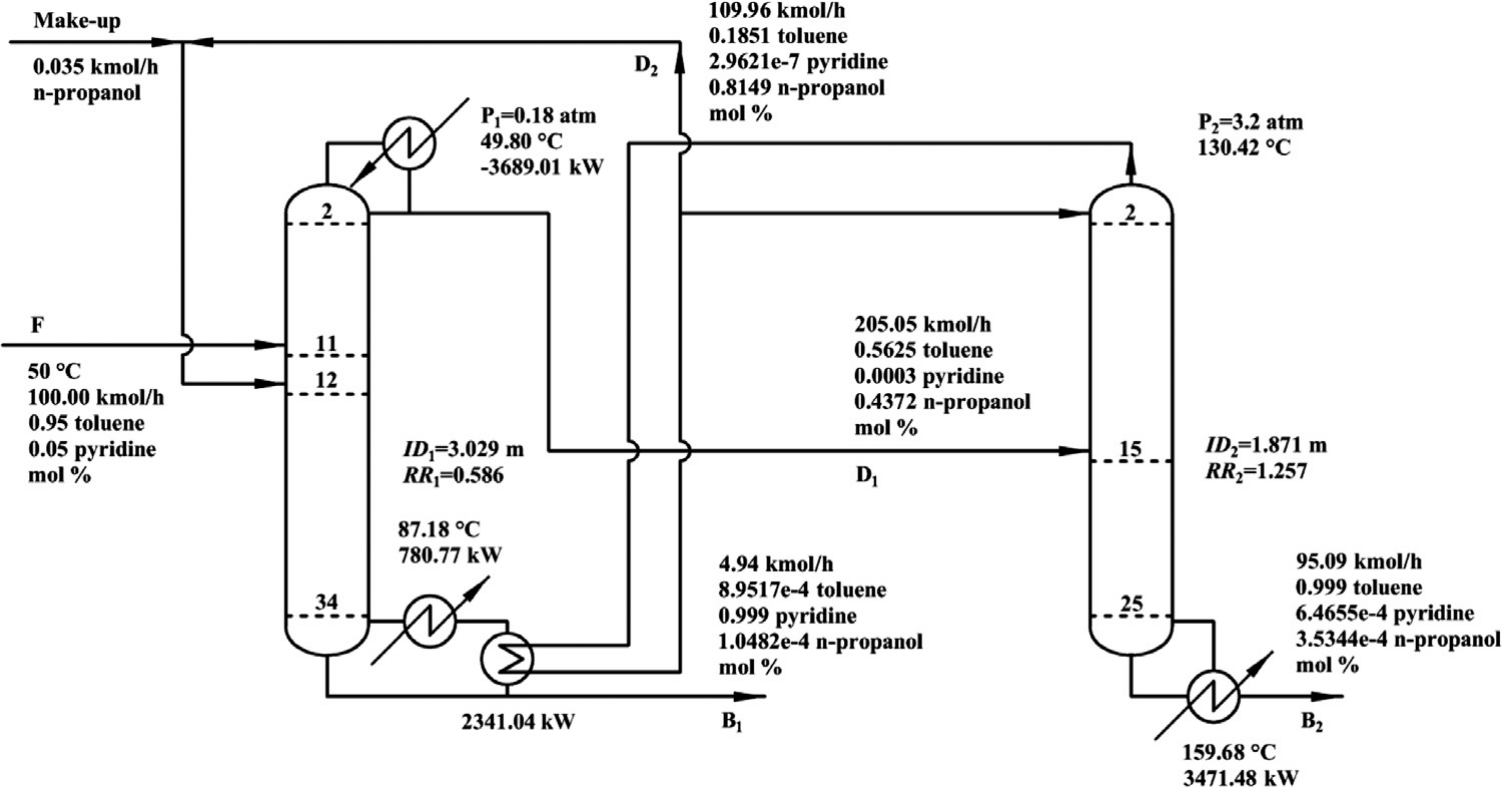
Flowsheet of EA-PSD for separating the toluene/pyridine mixture.

Fig. 35 shows flowsheet of EA-PSD for separating the toluene/pyridine mixture.

**Fig. 36:**
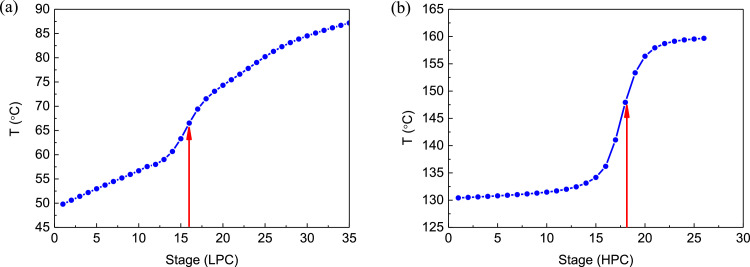
Temperature profiles of EA-PSD for separating the toluene/pyridine mixture: (a) the LPC and (b) the HPC.

Fig. 36 shows temperature profiles of EA-PSD for separating the toluene/pyridine mixture: (a) the LPC and (b) the HPC.

**Fig. 37:**
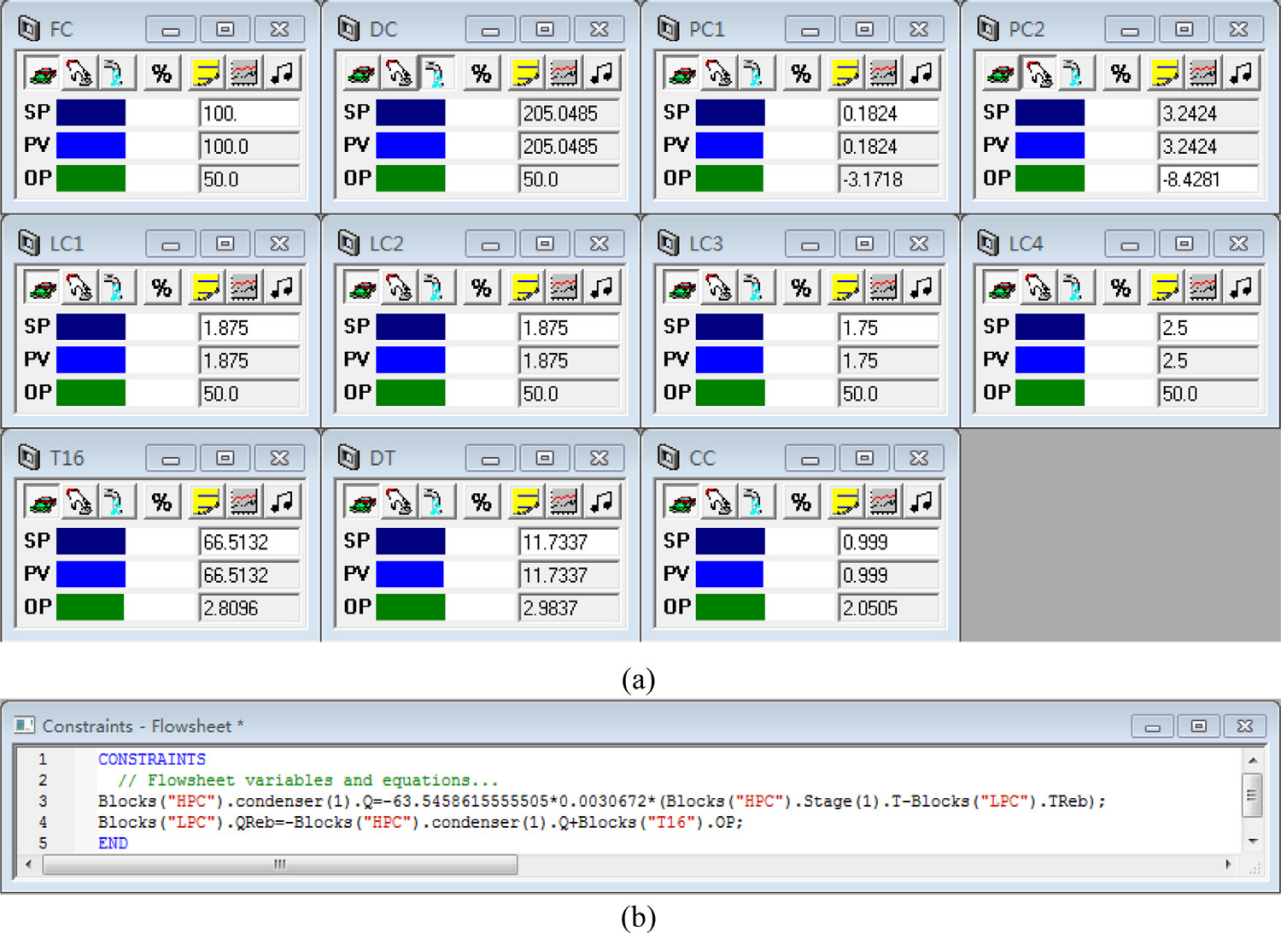
The proposed control structure with DTC of EA-PSD for separating the toluene/pyridine mixture: (a) control faceplate and (b) flowsheet equations.

Fig. 37 shows the proposed control structure with DTC of EA-PSD for separating the toluene/pyridine mixture: (a) control faceplate and (b) flowsheet equations.

**Fig. 38:**
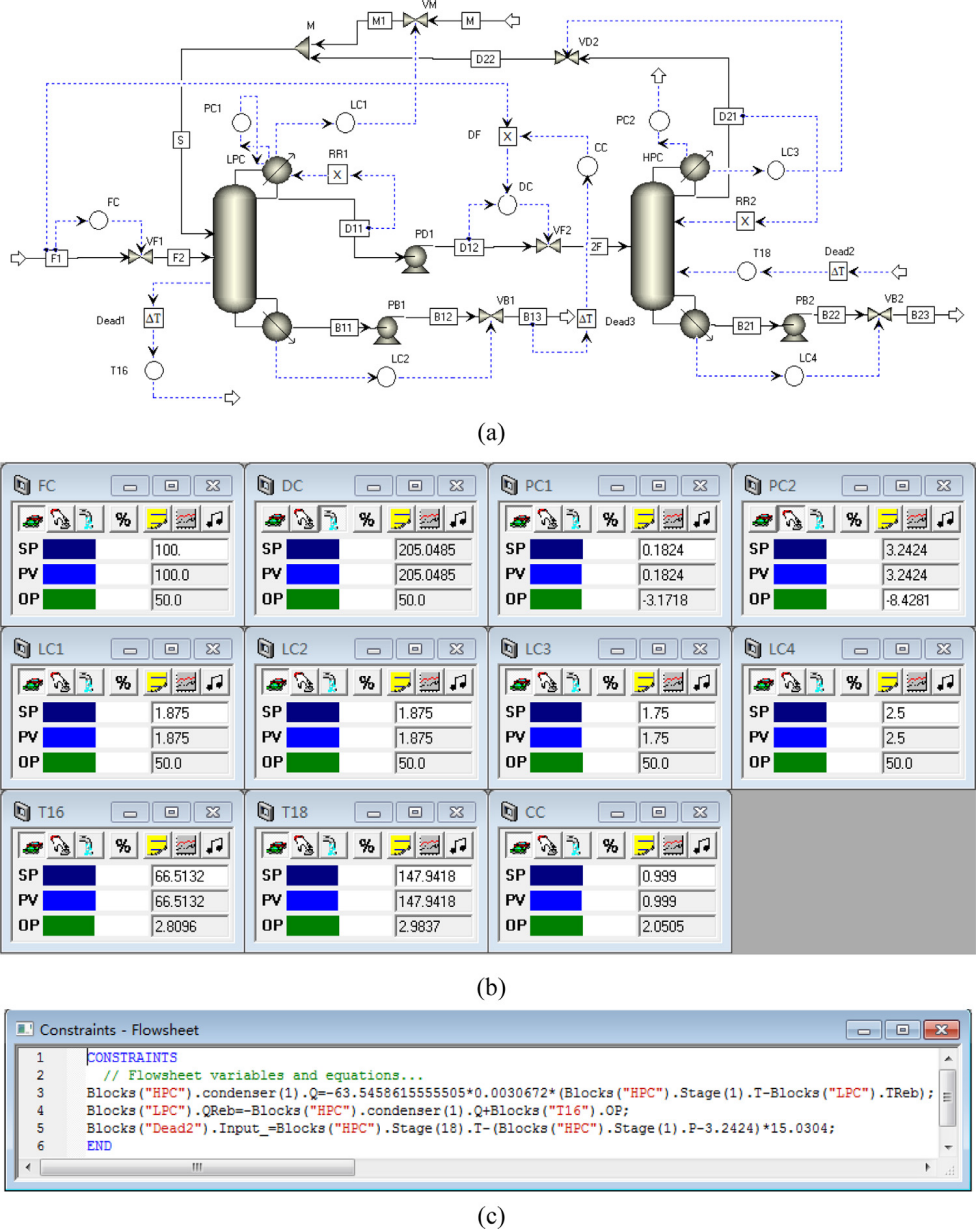
The proposed control structure with PCTC of EA-PSD for separating the toluene/pyridine mixture: (a) control flowsheet, (b) control faceplate and (c) flowsheet equations.

Fig. 38 shows the proposed control structure with PCTC of EA-PSD for separating the toluene/pyridine mixture: (a) control flowsheet, (b) control faceplate and (c) flowsheet equations.

**Fig. 39:**
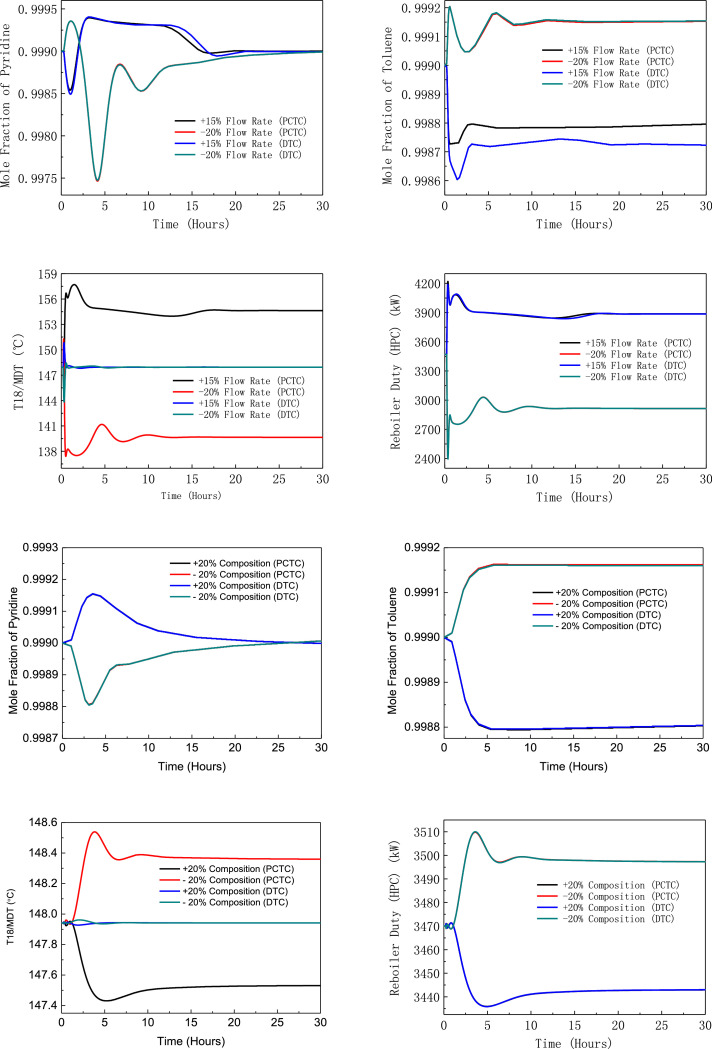
Dynamic performances of EA-PSD for separating the toluene/pyridine mixture under large feed disturbances.

Fig. 39 shows dynamic performances of EA-PSD for separating the toluene/pyridine mixture under large feed disturbances.

**Fig. 40:**
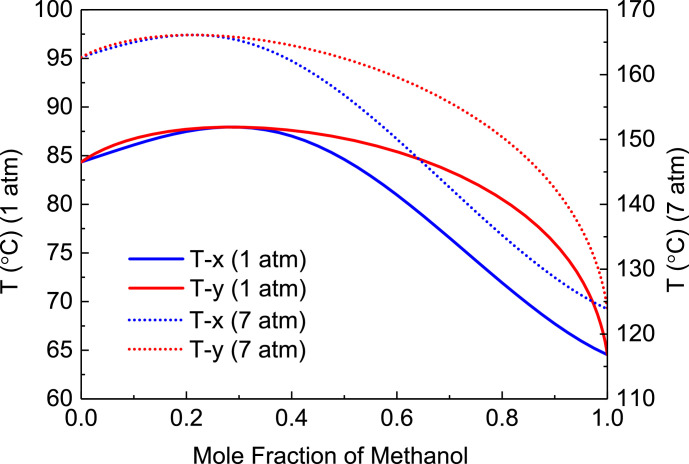
T-*xy* diagram of methanol/trimethoxysilane.

Fig. 40 shows T-xy diagram of methanol/trimethoxysilane.

**Fig. 41:**
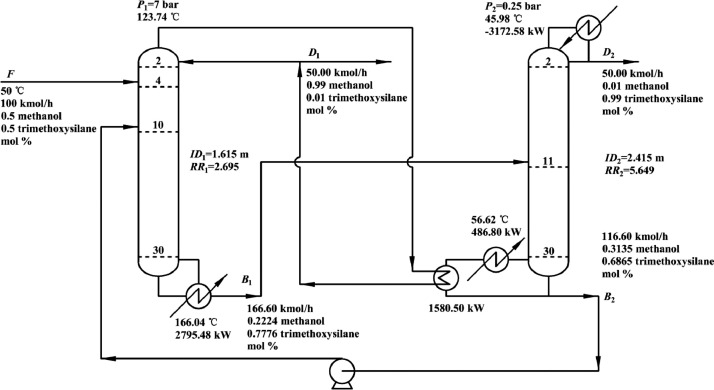
Flowsheet of the HPC->LPC sequence of PHI-PSD for separating the methanol/trimethoxysilane mixture.

Fig. 41 shows flowsheet of the HPC->LPC sequence of PHI-PSD for separating the methanol/trimethoxysilane mixture.

**Fig. 42:**
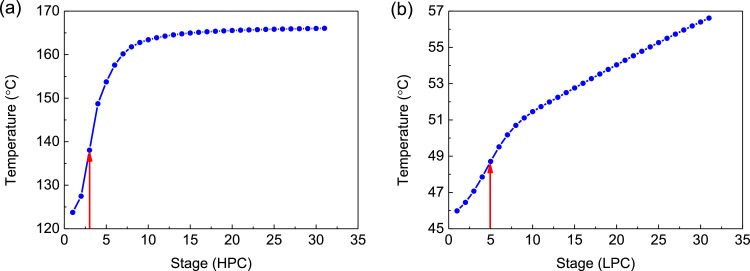
Temperature profiles of the HPC->LPC sequence of PHI-PSD for separating the methanol/trimethoxysilane mixture: (a) the HPC and (b) the LPC.

Fig. 42 shows temperature profiles of the HPC->LPC sequence of PHI-PSD for separating the methanol/trimethoxysilane mixture: (a) the HPC and (b) the LPC.

**Fig. 43:**
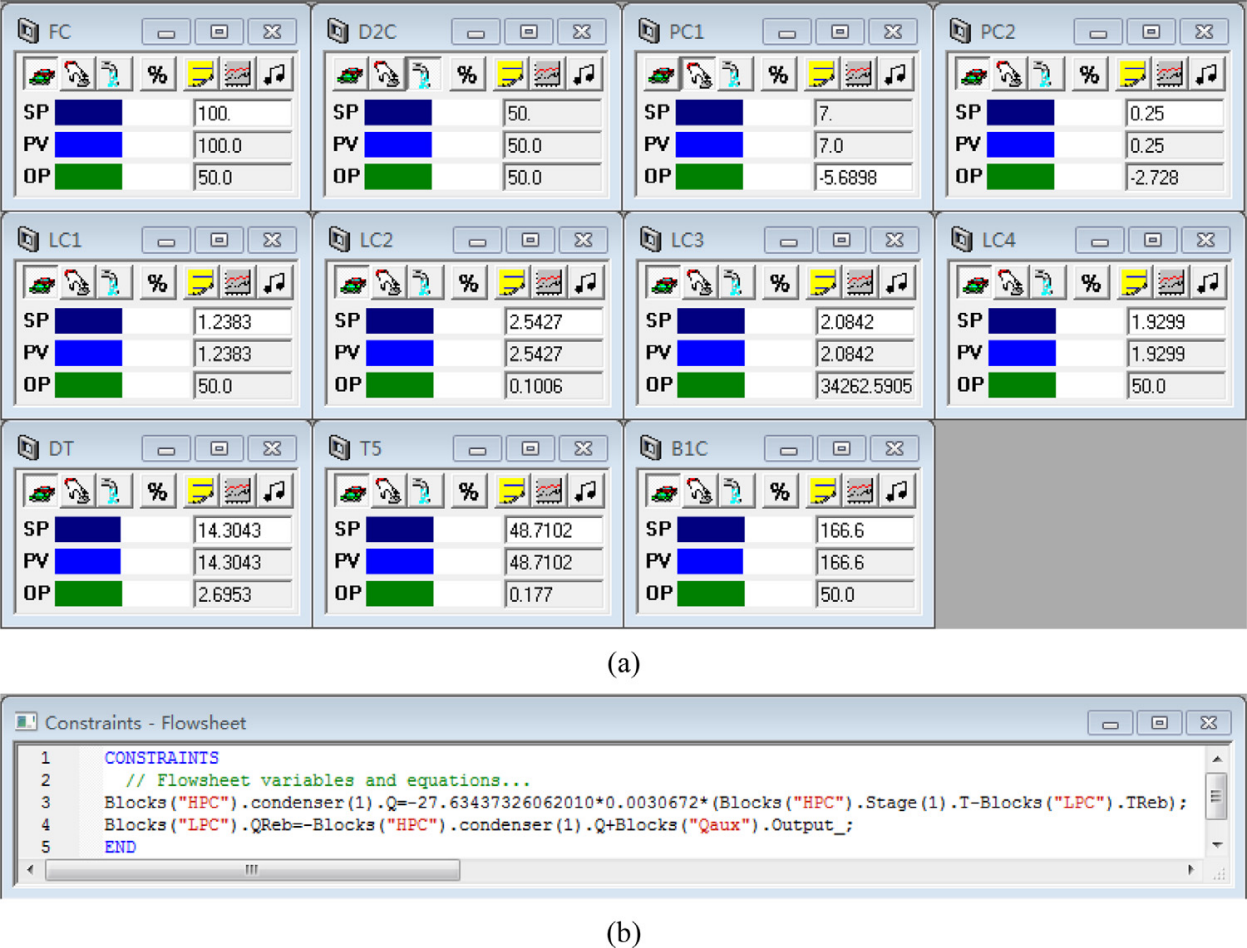
The proposed control structure with DTC of the HPC->LPC sequence of PHI-PSD for separating the methanol/trimethoxysilane mixture: (a) control faceplate and (b) flowsheet equations.

Fig. 43 shows the proposed control structure with DTC of the HPC->LPC sequence of PHI-PSD for separating the methanol/trimethoxysilane mixture: (a) control faceplate and (b) flowsheet equations.

**Fig. 44:**
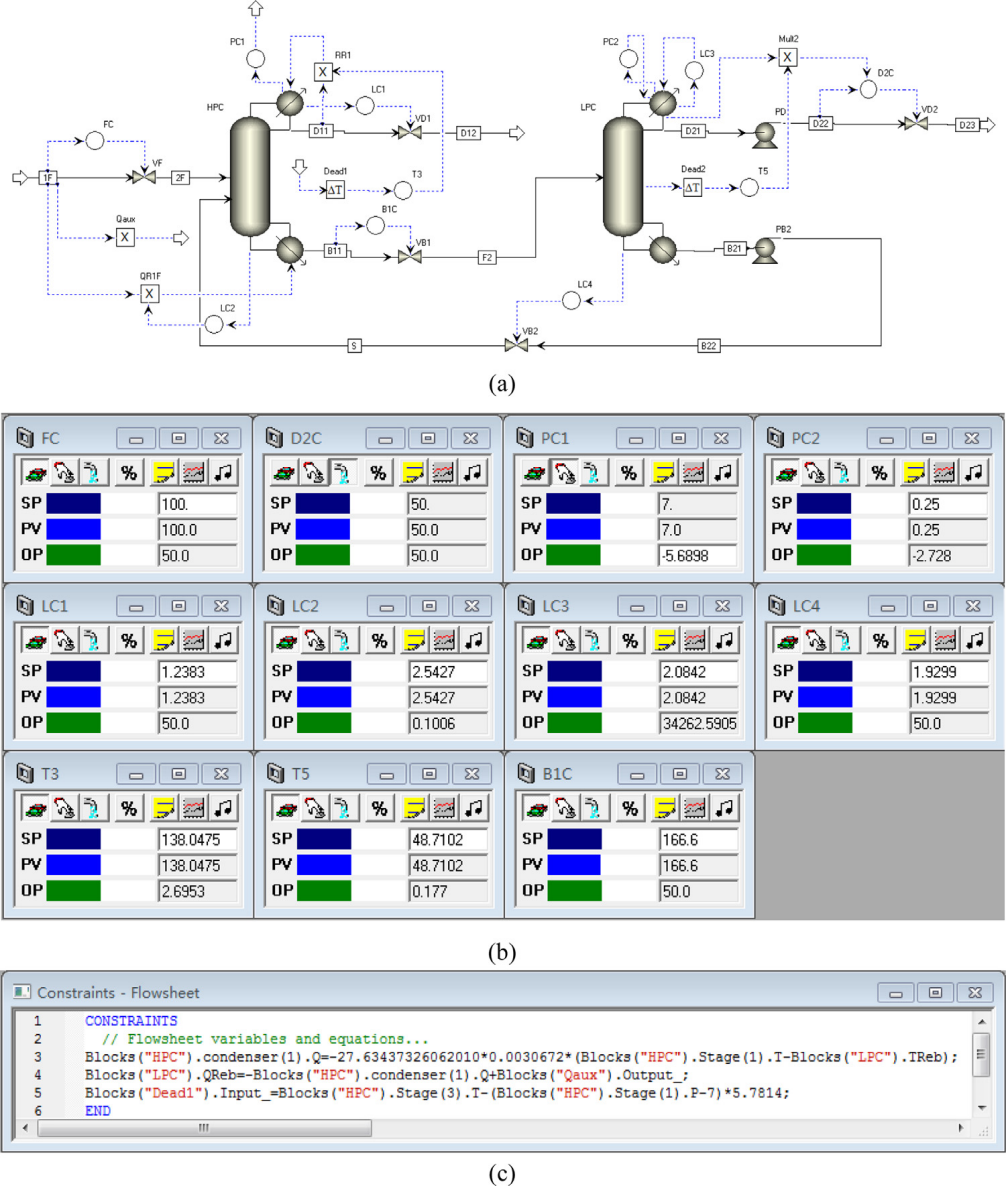
The proposed control structure with PCTC of the HPC->LPC sequence of PHI-PSD for separating the methanol/trimethoxysilane mixture: (a) control flowsheet, (b) control faceplate and (c) flowsheet equations.

Fig. 44 shows the proposed control structure with PCTC of the HPC->LPC sequence of PHI-PSD for separating the methanol/trimethoxysilane mixture: (a) control flowsheet, (b) control faceplate and (c) flowsheet equations.

**Fig. 45:**
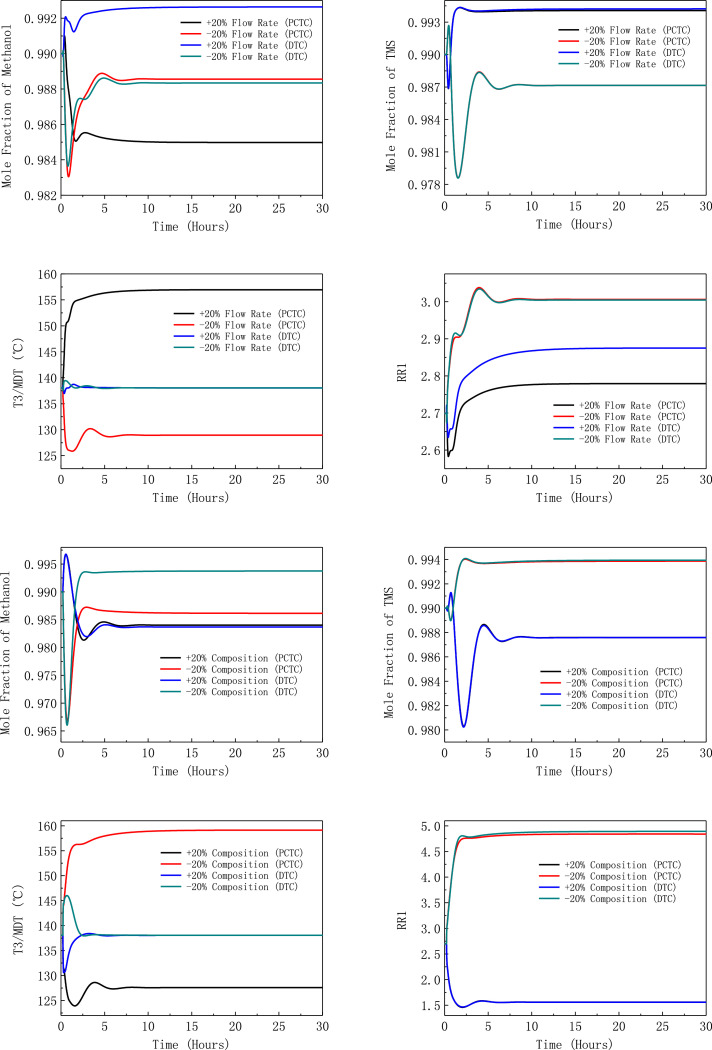
Dynamic performances of the HPC->LPC sequence of PHI-PSD for separating the methanol/trimethoxysilane mixture under large feed disturbances.

Fig. 45 shows dynamic performances of the HPC->LPC sequence of PHI-PSD for separating the methanol/trimethoxysilane mixture under large feed disturbances.

**Fig. 46:**
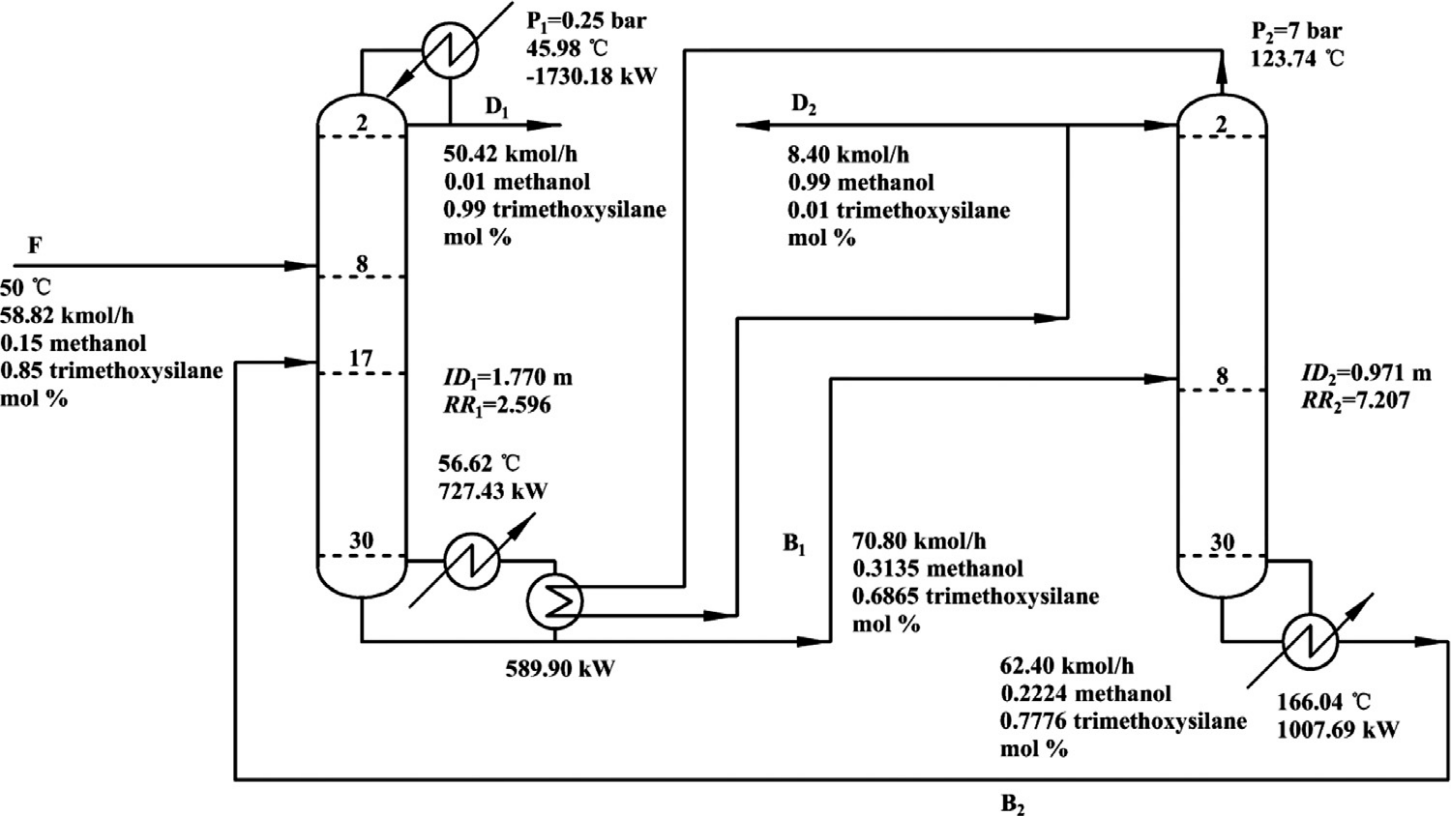
Flowsheet of the LPC->HPC sequence of PHI-PSD for separating the methanol/trimethoxysilane mixture.

Fig. 46 shows flowsheet of the LPC->HPC sequence of PHI-PSD for separating the methanol/trimethoxysilane mixture.

**Fig. 47:**
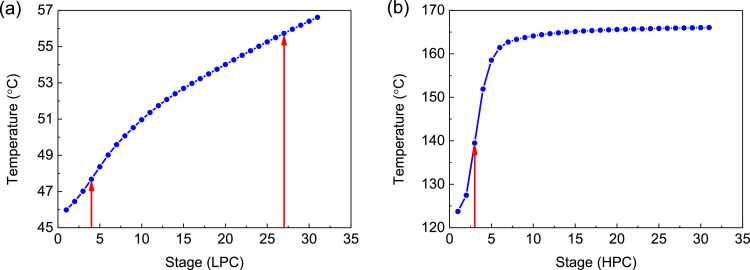
Temperature profiles of the LPC->HPC sequence of PHI-PSD for separating the methanol/trimethoxysilane mixture: (a) the LPC and (b) the HPC.

Fig. 47 shows temperature profiles of the LPC->HPC sequence of PHI-PSD for separating the methanol/trimethoxysilane mixture: (a) the LPC and (b) the HPC.

**Fig. 48:**
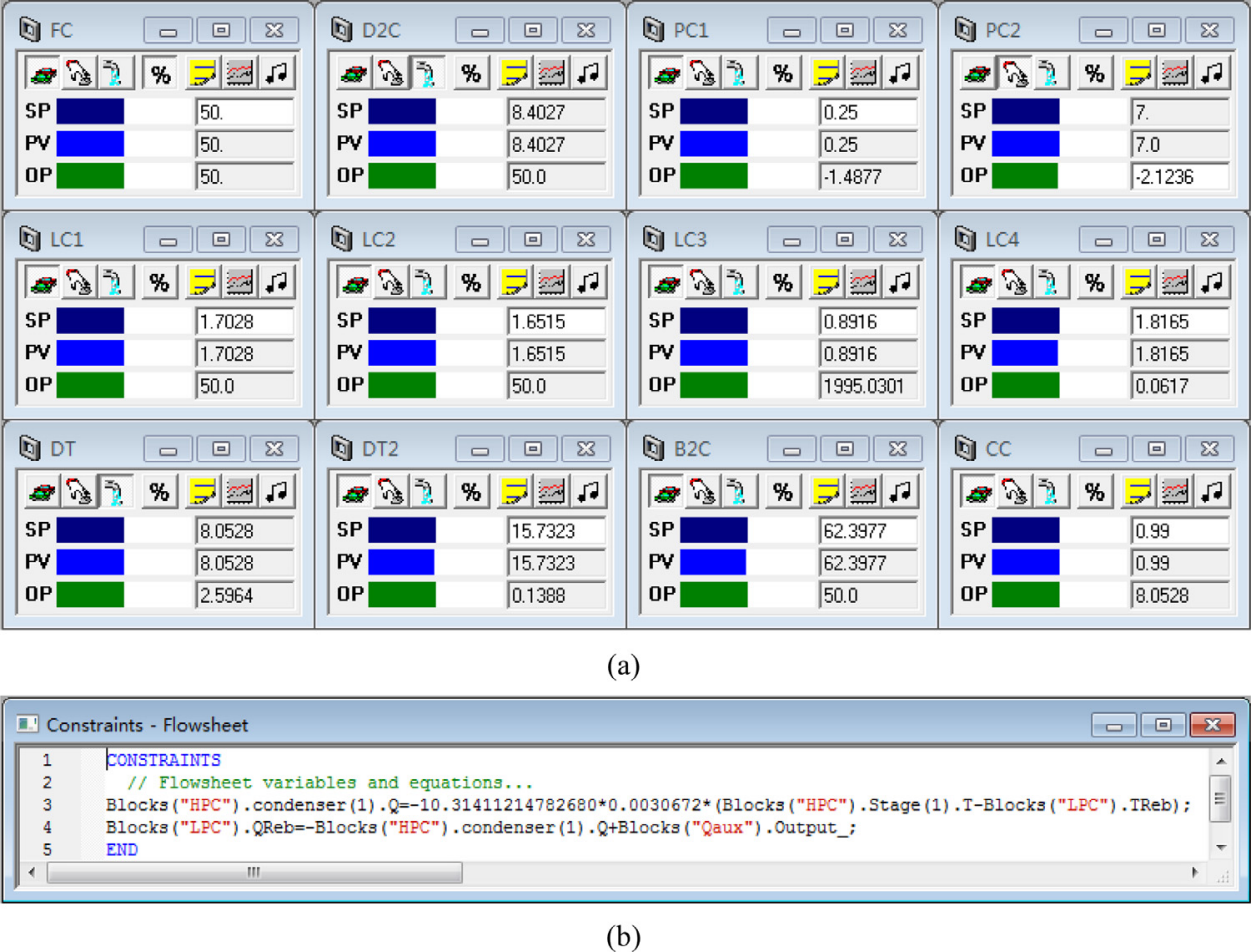
The proposed control structure with DTC of the LPC->HPC sequence of PHI-PSD for separating the methanol/trimethoxysilane mixture: (a) control faceplate and (b) flowsheet equations.

Fig. 48 shows the proposed control structure with DTC of the LPC->HPC sequence of PHI-PSD for separating the methanol/trimethoxysilane mixture: (a) control faceplate and (b) flowsheet equations.

**Fig. 49:**
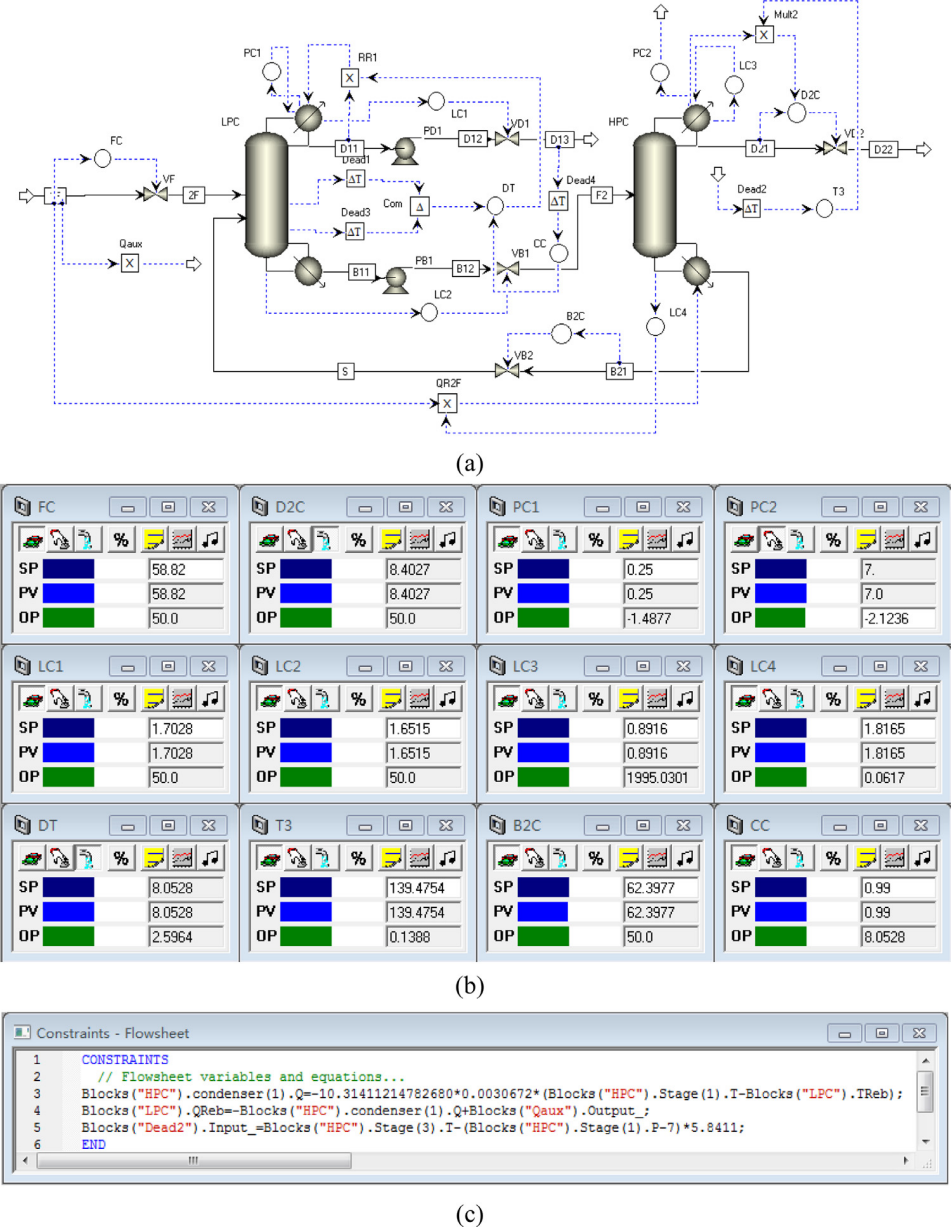
The proposed control structure with PCTC of the LPC->HPC sequence of PHI-PSD for separating the methanol/trimethoxysilane mixture: (a) control flowsheet, (b) control faceplate and (c) flowsheet equations.

Fig. 49 shows the proposed control structure with PCTC of the LPC->HPC sequence of PHI-PSD for separating the methanol/trimethoxysilane mixture: (a) control flowsheet, (b) control faceplate and (c) flowsheet equations.

**Fig. 50:**
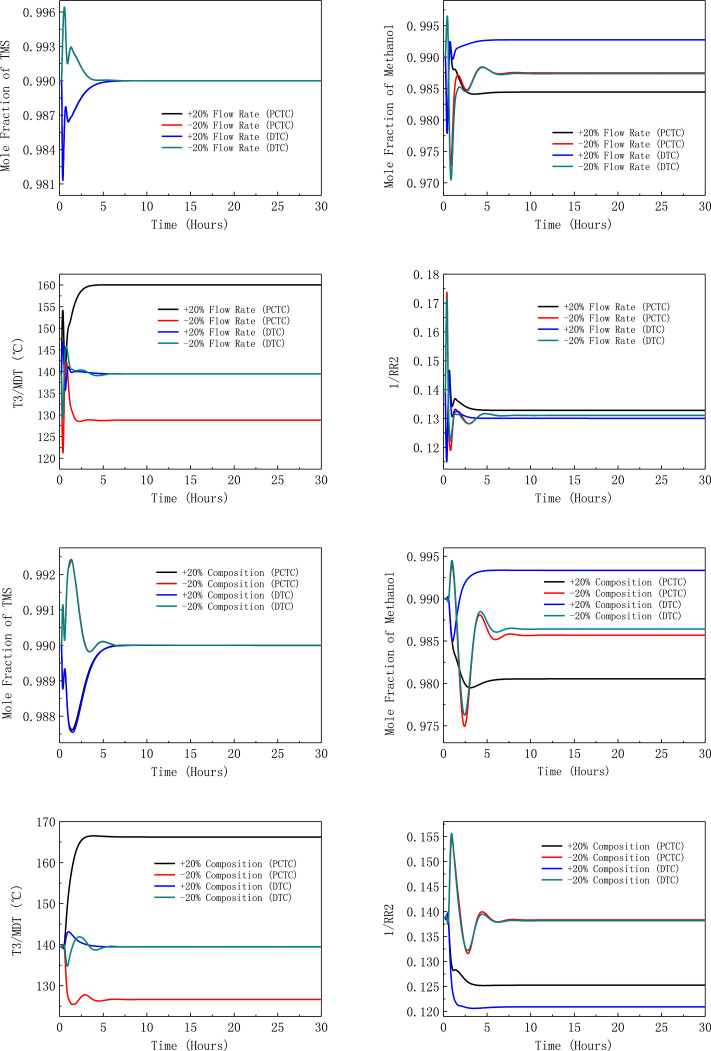
Dynamic performances of the LPC->HPC sequence of PHI-PSD for separating the methanol/trimethoxysilane mixture under large feed disturbances.

Fig. 50 shows dynamic performances of the LPC->HPC sequence of PHI-PSD for separating the methanol/trimethoxysilane mixture under large feed disturbances.

Section.1 Common information

The column bases and reflux drums are sized to provide 10 min of liquid holdup when half full. All the controllers are PI controllers: the tuning constants of pressure controllers are the same with default values with a gain of 20 and an integral time of 12 min; the tuning constants of flow controllers are the conventional values with a gain of 0.5 and an integral time of 0.3 min; the tuning constants of level controllers are the conventional values with a gain of 2 and an integral time of 9999 min. The temperature and composition controllers are tuned by the Tyreus-Luyben method after relay-feedback tests. The dead times of the temperature controller and composition controller are 1 min and 3 min, respectively.

Section.2 PHI-PSD for separating the toluene/ethanol mixture (LPC->HPC sequence)

The control loops of the proposed control structure with DTC of PHI-PSD for separating the toluene/ethanol mixture are listed as below:

(1) The fresh feed is flow controlled.
(2) The operating pressure of the LPC is controlled by manipulating the heat removal rate of the condenser.
(3) The reflux ratio of the HPC is fixed.
(4) The reflux ratio of the LPC is fixed.
(5) The reflux drum level of the HPC is controlled by manipulating the distillate flow rate.
(6) The reflux drum level of the LPC is controlled by manipulating the distillate flow rate.
(7) The base level of the HPC is controlled by manipulating the bottom flow rate.
(8) The base level of the LPC is controlled by manipulating the bottom flow rate.
(9) Composition/temperature cascade control is used to control the 21st-stage temperature of the LPC (T21). The ethanol product purity is measured as the input of the composition controller, whose output is the setpoint of the temperature controller. The temperature controller is “on cascade”. T21 is controlled by manipulating the heat removal rate of the auxiliary reboiler.
(10) The differential temperature (DT) of the bottom and the 20th stage of the HPC is controlled by manipulating the ratio of the reboiler duty of the HPC to the feed flow rate.

Section.3 PHI-PSD for separating the acetone/methanol mixture (LPC->HPC sequence)

In the LPC, the 47th-stage temperature (T47) is selected to be controlled. In the HPC, the 55th-stage temperature (T55) is selected to be controlled for PCTC, while the difference between the bottom temperature and T55 is controlled for DTC. The diagram of operating pressure vs. differential temperature is shown in Fig. 10.

The control loops of the proposed control structure with DTC of PHI-PSD for separating the acetone/methanol mixture are listed as below:

(1) The fresh feed is flow controlled.
(2) The operating pressure of the LPC is controlled by manipulating the heat removal rate of the condenser.
(3) The reflux ratio of the HPC is fixed.
(4) The reflux ratio of the LPC is fixed.
(5) The reflux drum level of the HPC is controlled by manipulating the distillate flow rate.
(6) The reflux drum level of the LPC is controlled by manipulating the distillate flow rate.
(7) The base level of the HPC is controlled by manipulating the bottom flow rate.
(8) The base level of the LPC is controlled by manipulating the bottom flow rate.
(9) The 47th-stage temperature of the LPC (T47) is controlled by manipulating the heat removal rate of the auxiliary reboiler.
(10) The differential temperature (DT) of the bottom and the 55th stage of the HPC is controlled by manipulating the ratio of the reboiler duty of the HPC to the feed flow rate.

Section.4 FHI-PSD for separating the toluene/ethanol mixture (LPC->HPC sequence)

The control loops of the proposed control structure with DTC of FHI-PSD for separating the toluene/ethanol mixture are listed as below:

(1) The fresh feed is flow controlled.
(2) The operating pressure of the LPC is controlled by manipulating the heat removal rate of the condenser.
(3) The reflux ratio of the HPC is fixed.
(4) The reflux drum level of the HPC is controlled by manipulating the distillate flow rate.
(5) The reflux drum level of the LPC is controlled by manipulating the distillate flow rate.
(6) The base level of the HPC is controlled by manipulating the bottom flow rate.
(7) The base level of the LPC is controlled by manipulating the bottom flow rate.
(8) The 11th-stage temperature of the LPC (T11) is controlled by manipulating the reflux ratio.
(9) The differential temperature (DT) of the bottom and the 19th stage of the HPC is controlled by manipulating the ratio of the reboiler duty of the HPC to the feed flow rate.

Section.5 FHI-PSD for separating the methanol/chloroform mixture (LPC->HPC sequence)

In the LPC, the 19th-stage temperature (T19) is selected to be controlled. In the HPC, the 23rd-stage temperature (T23) is selected to be controlled for PCTC, while the difference between the bottom temperature and T23 is controlled for DTC. The diagram of operating pressure vs. differential temperature is shown in Fig. 23.

The control loops of the proposed control structure with DTC of FHI-PSD for separating the methanol/chloroform mixture are listed as below:

(1) The fresh feed is flow controlled.
(2) The operating pressure of the LPC is controlled by manipulating the heat removal rate of the condenser.
(3) The reflux ratio of the HPC is fixed.
(4) The reflux drum level of the HPC is controlled by manipulating the distillate flow rate.
(5) The reflux drum level of the LPC is controlled by manipulating the distillate flow rate.
(6) The base level of the HPC is controlled by manipulating the bottom flow rate.
(7) The base level of the LPC is controlled by manipulating the bottom flow rate.
(8) The 19th-stage temperature of the LPC (T19) is controlled by manipulating the reflux ratio.
(9) The differential temperature (DT) of the bottom and the 23rd stage of the HPC is controlled by manipulating the ratio of the reboiler duty of the HPC to the feed flow rate.

Section.6 FHI-PSD for separating the ethanol/ethyl acetate mixture (HPC->LPC sequence)

The control loops of the proposed control structure with DTC of FHI-PSD for separating the ethyl acetate/ethanol mixture are listed as below:

(1) The fresh feed is flow controlled.
(2) The operating pressure of the LPC is controlled by manipulating the heat removal rate of the condenser.
(3) The reflux ratio of the HPC is fixed.
(4) The reflux drum level of the HPC is controlled by manipulating the distillate flow rate.
(5) The reflux drum level of the LPC is controlled by manipulating the distillate flow rate.
(6) The base level of the HPC is controlled by manipulating the bottom flow rate.
(7) The base level of the LPC is controlled by manipulating the bottom flow rate.
(8) The 25th-stage temperature of the LPC (T25) is controlled by manipulating the reflux ratio.
(9) The differential temperature (DT) of the bottom and the 50th stage of the HPC is controlled by manipulating the ratio of the reboiler duty of the HPC to the feed flow rate.

Section.7 EA-PSD for separating the toluene/pyridine mixture

The control loops of the proposed control structure with DTC of EA-PSD for separating the toluene/pyridine mixture are listed as below:

(1) The fresh feed is flow controlled.
(2) The distillate flow rate of the LPC is proportional to the feed flow rate, with the proportion being adjusted by a pyridine product composition controller.
(3) The operating pressure of the LPC is controlled by manipulating the heat removal rate of the condenser.
(4) The reflux ratio of the HPC is fixed.
(5) The reflux ratio of the LPC is fixed.
(6) The reflux drum level of the HPC is controlled by manipulating the distillate flow rate.
(7) The reflux drum level of the LPC is controlled by manipulating the makeup flow rate.
(8) The base level of the HPC is controlled by manipulating the bottom flow rate.
(9) The base level of the LPC is controlled by manipulating the bottom flow rate.
(10) The 16th-stage temperature of the LPC (T16) is controlled by manipulating the heat duty of the auxiliary reboiler.
(11) The differential temperature (DT) of the bottom and the 18th stage of the HPC is controlled by manipulating the reboiler duty.

Section.8 PHI-PSD for separating the methanol/trimethoxysilane mixture (HPC->LPC sequence)

The control loops of the proposed control structure with DTC of the HPC->LPC sequence of PHI-PSD for separating the methanol/trimethoxysilane mixture are listed as below:

(1) The fresh feed is flow controlled.
(2) The operating pressure of the LPC is controlled by manipulating the heat removal rate of the condenser.
(3) The reflux drum level of the HPC is controlled by manipulating the distillate flow rate.
(4) The reflux drum level of the LPC is controlled by manipulating the reflux flow rate.
(5) The base level of the LPC is controlled by manipulating the bottom flow rate.
(6) The reboiler duty of the HPC is proportional to the feed flow rate. This ratio is manipulated to control the base level of the HPC.
(7) The bottom flow of the HPC is flow controlled at a constant initial value.
(8) The auxiliary reboiler duty is proportional to the feed flow rate.
(9) The differential temperature (DT) of the 3rd stage and the top of the HPC is controlled by manipulating the reflux ratio.
(10) The 5th-stage temperature of the LPC (T5) is controlled by manipulating the reciprocal of the reflux ratio.

Section.9 PHI-PSD for separating the methanol/trimethoxysilane mixture (LPC->HPC sequence)

The control loops of the proposed control structure with DTC of the LPC->HPC sequence of PHI-PSD for separating the methanol/trimethoxysilane mixture are listed as below:

(1) The fresh feed is flow controlled.
(2) The operating pressure of the LPC is controlled by manipulating the heat removal rate of the condenser.
(3) The reflux drum level of the LPC is controlled by manipulating the distillate flow rate.
(4) The reflux drum level of the HPC is controlled by manipulating the reflux flow rate.
(5) The base level of the LPC is controlled by manipulating the bottom flow rate.
(6) The base level of the HPC is controlled by manipulating the ratio of the reboiler duty of the HPC to the feed flow rate.
(7) The bottom flow rate of the HPC is flow controlled at a constant initial value.
(8) The auxiliary reboiler duty is proportional to the feed flow rate.
(9) The differential temperature (DT2) of the 3rd stage and the top of the HPC is controlled by manipulating the reciprocal of the reflux ratio.
(10) Composition/temperature cascade control is used to control the differential temperature (DT) of the 4th stage and the 27th stage of the LPC. Trimethoxysilane product purity is measured as the input of the composition controller, whose output is the setpoint of the temperature controller. The temperature controller is “on cascade”. DT is controlled by manipulating the reflux ratio.

Section.10 Integral absolute error (IAE)

Comparison of IAE of important variables between PCTC and DTC under large feed disturbances is shown in Table 19. If 20% feed disturbance cannot be controlled, 15% feed disturbance is introduced. If 15% feed disturbance cannot be controlled, the values are not presented as the results of 10% feed disturbance have been shown.

Section.11 Other information

## 2 Experimental design, materials and methods

The simulations are implemented by Aspen Plus and Aspen Dynamics. Aspen Plus is used to establish steady-state designs. After steady-state designs are established, they are converted into dynamic processes. The dynamic research is implemented in Aspen Dynamics.

## Declaration of Competing Interest

The authors declare that they have no known competing financial interests or personal relationships which have, or could be perceived to have, influenced the work reported in this article.
